# A randomized controlled trial of Roux-en-Y gastrojejunostomy vs. gastroduodenostomy with respect to the improvement of type 2 diabetes mellitus after distal gastrectomy in gastric cancer patients

**DOI:** 10.1371/journal.pone.0188904

**Published:** 2017-12-07

**Authors:** Yoon Young Choi, Sung Hoon Noh, Ji Yeong An

**Affiliations:** 1 Department of Surgery, Yonsei University College of Medicine, Seoul, Korea; 2 Department of Surgery, Samsung Medical Center, Sungkyunkwan University School of Medicine, Seoul, Korea; Weill Cornell Medical College Qatar, QATAR

## Abstract

The purpose of this study is to compare the effect of diabetes control induced by Roux-en-Y gastrojejunostomy(RY) vs Billroth-I reconstruction(BI) after distal gastrectomy in patients with early gastric cancer(EGC) and type 2 diabetes(T2DM). Forty EGC patients with T2DM, aged 20–80 years, who were expected to undergo curative distal gastrectomy were randomized 1:1 to RY(n = 20) or BI(n = 20). Diabetes medication status, biochemical and hormonal data including blood glucose, HbA1c, insulin, C-peptide, HOMA-IR, ghrelin, leptin, GLP-1, PYY, and GIP were evaluated for 12 months after surgery. Although pre- and postoperative 12-month fasting and postprandial glucose levels did not show a significant difference, HbA1c, C-peptide, and HOMA-IR levels were significantly improved at 12 months after surgery in both BI and RY groups. Sixty percent of RY patients and 20% of BI patients decreased their medication satisfying FBS<126 mg/dL and HbA1c<6.5% and 5% of BI patients stopped their medication satisfying the criteria of FBS<126 mg/dL and HbA1c<6.0%. The improvement patterns were more sustainable with less fluctuation in RY than in BI. On hormonal analysis, ghrelin and leptin levels were decreased and PYY and GIP levels were increased at 12 months after surgery in both groups without significant difference according to the reconstruction type and diabetic improvement status except ghrelin. In gastric cancer surgery, RY reconstruction showed better and more durable diabetes control compared to BI during the first year after surgery. Gastric cancer surgery led to decreased ghrelin and leptin and increased PYY and GIP, which might have a role in improving insulin resistance and glucose homeostasis.

## Introduction

Diabetes mellitus (DM) is one of the most important health problems worldwide. Because DM is a multi-factorial chronic, progressive disease with a gradual increase in insulin resistance and beta cell failure, intensification of medical treatment is often required. [1] Although the prevalence of DM in gastric cancer patients has not been reported, it may be similar to that in the general population, and the negative effect of DM on quality of life is also applicable to gastric cancer patients. Considering the increasing proportion of EGC and improved survival, simultaneous diabetes control in addition to cure of cancer has become more important.

After gastric cancer surgery, most patients experience reduced food intake, weight loss, and intestinal malabsorption, and some of them show improvement in metabolic diseases such as hyperglycemia, hypercholesterolemia, and hypertension. Although the purpose of metabolic surgery and gastric cancer surgery is different, there is a clinical and technical connection between the two procedures. Therefore, in our previous study, we highlighted the positive effect of conventional gastrectomy on diabetes control in patients with type 2 DM (T2DM) and EGC. [2] In Korea, the main reconstruction method after distal gastrectomy for gastric cancer surgery is gastroduodenostomy (Billroth I reconstruction [BI]). When it is difficult to perform BI because of the tumor location or resection extent, gastrojejunostomy (Billroth II reconstruction [BII]) or Roux-en-Y gastrojejunostomy (RY) is performed (S1 Fig). We previously investigated the short-term effect of BI and BII as routine gastric cancer surgeries on T2DM, and found that BII, which involves bypass of the short segment of proximal small bowel, did not provide significant additional benefits in terms of glucose metabolism compared to BI reconstruction. [2] Considering the antidiabetic effect of RY gastric bypass used in bariatric and metabolic surgery, RY reconstruction in gastric cancer surgery may provide better glucose metabolism than BI and BII. [3] Therefore, in the present study, we attempted to investigate the impact of RY in distal gastrectomy, which can provide a longer bypass length of the proximal jejunum than BI and BII. Such an evaluation will allow the surgeons to select a favorable reconstruction type after gastrectomy in gastric cancer patients with diabetes.

## Materials and methods

### Study design and participants

This study was a prospective randomized study performed at Yonsei University Hospital from July 2011 to May 2014. Study participants were patients diagnosed with early gastric cancer and T2DM. It was possible to perform distal gastrectomy in all of the patients and they were taking diabetes medication before the diagnosis of gastric cancer. Patients were eligible for this study if they were between 20 and 80 years of age and were expected to undergo curative distal gastrectomy for distal gastric adenocarcinoma. Patients with the following conditions were excluded: (a) other malignancies; (b) preoperative chemotherapy; (c) other endocrine disorders such as thyroid or adrenal disease; (d) moderate to severe cardiovascular, pulmonary or renal disease; (e) active infection; and (f) Vulnerable patients (pregnant women, children, cognitively impaired persons, etc.). If patients were plan to receive postoperative chemotherapy or suffered from immediate postoperative complications requiring reoperation, they were also excluded from analysis. This study was reviewed and approved by the Institutional Review Board of Severance Hospital, Yonsei University College of Medicine (IRB File No. 4-2011-0109), and written informed consent was obtained from all patients prior to operation. The study was registered at www.clinicaltrials.gov (NCT01375738).

### Randomization

Patients were randomly assigned at a 1:1 ratio to RY or to BI before surgery by using computer-generated randomization table. Because our previous study showed that the duration of DM was a significant predictive factor that was associated with postoperative diabetes control, diabetic duration, > 5 years vs ≤ 5 years, was used as a stratification factor. Neither patients nor investigators were masked to treatment assignment.

### Surgical procedures

In RY reconstruction, [4] the duodenum was transected 1–2 cm distal to the pyloric ring using a linear stapler and gastric resection was done using two linear staplers. For reconstruction, the jejunum was divided at 25 cm distal to the ligament of Treitz and a hole was made on the joining end of the stapling line and the greater curvature of the stomach using ultrasonic shears. The transected distal jejunum was brought up using the antecolic method, and a hole was made 7 cm distal to the jejunal transection line. A gastrojejunostomy was made using a linear stapler, and the common entry hole was also closed with a linear stapler. To perform the jejunojejunostomy, a hole was made on the anti-mesenteric border of the jejunum 30 cm distal to the gastrojejunostomy and another hole was made on the anti-mesenteric end of the stapling line of the proximal jejunum previously transected. Each arm of the endoscopic linear stapler was inserted into the proximal and distal jejunum, and a side-to-side anastomosis was performed.

In Billroth I reconstruction, [5] duodenal resection was performed immediately below the pyloric ring intracorporeally using an endoscopic linear stapler. After gastric resection with two linear staplers, a 45 mm endoscopic linear stapler was inserted through the left lower port and each jaw of the stapler was gently inserted in each gastric and duodenal hole. Following approximation of the posterior walls of the gastric remnant and the duodenum, the jaws of the stapler were closed and fired to create an anastomosis. The common entry hole was closed with two linear staplers. Other surgical processes except for reconstruction were similar to those described here for the RY group. Generally, D1+β or D2 lymph node dissection was performed according to the guidelines of the Japanese Gastric Cancer Association, [6] and all surgery was performed by laparoscopic approach, and truncal vagotomy was conducted in all cases.

During the admission period, all of the patients received nutrition education for adapting gastrectomy status, and they were educated to restrict high-carbohydrate, high-fat diet, and drinks with sugar. In the out-patient clinic, patients’ gastrointestinal symptoms such as nausea, vomiting, diarrhea, dyspepsia including symptoms related dumping syndrome were asked and checked.

### Study outcome evaluation

To evaluate the effect of Roux-en-Y gastrojejunostomy (RY group) compared to Billroth-I reconstruction (BI group) after distal gastrectomy in improving T2DM, clinical and biochemical data were collected during the preoperative period and at 3, 6, 9, and 12 months after gastrectomy. The clinical characteristics of the enrolled patients, including age, sex, body mass index (BMI), comorbidities, diabetes duration, diabetes medication, and perioperative outcomes, such as the extent of lymph node dissection, blood loss during surgery, length of hospital stay, and postoperative complications were recorded. Complications were recorded according to the Clavien-Dindo Classification of surgical complications.

Blood samples were obtained after an overnight fast. Patients visited the hospital preoperatively, and at 3, 6, 9, and 12 months after surgery for physical examination, laboratory tests, imaging, and/or endoscopy. The variables for evaluating the status of glucose control included body weight, body mass index, biochemical data including fasting glucose (FBS) and postprandial 2 hour glucose (PP2), insulin, and C-peptide, HbA1c, homeostasis model assessment-estimated insulin resistance (HOMA-IR), and changes in the diabetes medication status. For gut hormone analysis, we checked the levels of active glucagon-like peptide-1 (GLP-1), glucose-dependent insulinotropic polypeptide (GIP), Peptide YY (PYY), acylated ghrelin and leptin in the fasting state.

The degree of diabetes control was divided into the following three groups:[2]

1. Remission: No medication and fasting blood glucose (FBS) < 126 mg/dL and HbA1c <6.0%.
2. Improved: Reduced medication and FBS <126 mg/dL and HbA1c <6.5%.
3. Stationary: No change in medication, or patients excluded from the improved and remission categories.

### Hormonal analysis

#### Ghrelin (active)

This assay is a Sandwich ELISA based on: capture of human ghrelin molecules (active form) in the sample by anti-human ghrelin IgG and immobilization of the resulting complex to the wells of a microtiter plate coated by a pre-titered amount of anchor antibodies, and the simultaneous binding of a second biotinylated antibody to ghrelin, wash away of unbound materials, followed by conjugation of horseradish peroxidase to the immobilized biotinylated antibodies, wash away of free enzyme, and quantification of immobilized antibody-enzyme conjugates by monitoring horseradish peroxidase activities in the presence of the substrate 3,3’,5,5’-tetra-methylbenzidine. The enzyme activity is measured spectrophotometrically by the increased absorbency at 450 nm, corrected from the absorbency at 590 nm, after acidification of formed products. Since the increase in absorbency is directly proportional to the amount of captured human ghrelin (active form) in the unknown sample, the concentration of active ghrelin can be derived by interpolation from a reference curve generated in the same assay with reference standards of known concentrations of human ghrelin.

#### Leptin

This assay is a direct Sandwich ELISA based, sequentially, on: capture of human leptin by a polyclonal rabbit anti-human leptin antibody immobilized on a 96-well microtiter plate, wash away unbound materials, binding of a biotinylated monoclonal antibody to the captured human leptin, wash away unbound materials, binding of streptavidin-horseradish peroxidase to the immobilized biotinylated antibodies, wash away free enzyme conjugates, and quantification of bound streptavidin-horseradish peroxidase with the substrate 3,3’,5,5’-tetramethylbenzidine.The enzyme activity is measured spectrophotometrically by the increased absorbency at 450 nm—590 nm after acidification of formed products. Since the increase in absorbency is directly proportional to the amount of captured human leptin in the unknown sample, the latter can be derived by interpolation from a reference curve generated in the same assay with reference standards of known concentrations of human leptin.

#### GLP_1 active (glucagon-like peptide-1)

This assay is based, sequentially, on: capture of active GLP-1 from sample by a monoclonal antibody, immobilized in the wells of a microwell plate, that binds specifically to the N-terminal region of active GLP-1 molecule, washing to remove unbound materials, binding of an anti GLP-1-alkaline phosphatase detection conjugate to the immobilized GLP-1, washing off unbound conjugate, and quantification of bound detection conjugate by adding MUP (methyl umbelliferyl phosphate) which in the presence of alkaline phosphatase forms the fluorescent product umbelliferone. Since the amount of fluorescence generated is directly proportional to the concentration of active GLP-1 in the unknown sample, the latter can be derived by interpolation from a reference curve generated in the same assay with reference standards of known concentrations of active GLP-1.

#### PYY (peptide YY)

This assay is a Sandwich ELISA based on: capture of PYY in the sample by anti-human PYY IgG and immobilization of the resulting complex to the wells of a microtiter plate coated by a pre-titered amount of anchor antibodies, binding of a second biotinylated antibody to PYY after brief washing, wash away of unbound materials, followed by conjugation of horseradish peroxidase to the immobilized biotinylated antibodies, wash away of free enzyme, and quantification of immobilized antibody-enzyme conjugates by monitoring horseradish peroxidase activities in the presence of the substrate 3,3’,5,5’-tetra-methylbenzidine. The enzyme activity is measured spectrophotometrically by the increased absorbency at 450 nm, corrected from the absorbency at 590 nm, after acidification of formed products. Since the increase in absorbency is directly proportional to the amount of captured PYY in the unknown sample, the concentration of PYY can be derived by interpolation from a reference curve generated in the same assay with reference standards of known concentrations of Human PYY.

#### GIP total (Gastric inhibitory polypeptide, glucose-dependent insulinotropic polypeptide)

This assay is a Sandwich ELISA based, sequentially, on: capture of human GIP molecules from samples to the wells of a microtiter plate coated by a pre-titered amount of anti-GIP monoclonal antibodies, wash away of unbound materials from samples, binding of a second biotinylated anti-GIP polyclonal antibody to the captured molecules, wash away of unbound materials from samples, incubation of streptavidin-horseradish peroxidase conjugate to bind to the immobilized biotinylated antibodies, wash away of free enzyme conjugates, and quantification of immobilized antibody-enzyme conjugates by monitoring horseradish peroxidase activities in the presence of the substrate 3,3’,5,5’-tetramethylbenzidine. The enzyme activity is measured spectrophotometrically by the increased absorbency at 450 nm, corrected from the absorbency at 590 nm, after acidification of formed products. Since the increase in absorbency is directly proportional to the amount of captured human GIP in the unknown sample, the latter can be derived by interpolation from a reference curve generated in the same assay with reference standards of known concentrations of human GIP.

### Statistical analysis

The sample size was calculated based on our previous results that were published in gastric cancer patients with T2DM undergoing gastrectomy. [2,3] In our previous study, about 15% of patients satisfied the improvement criteria (reduced medication and FBS <126 mg/dL and HbA1c <6.5%) after distal gastrectomy with BI reconstruction. Assuming the probability of a type I error (α) of 0.05 (two-sided) and a power of 80%, we estimated that we would need a sample size of 18 patients per arm to detect a difference of 40% between BI and RY group in the improvement of DM at 12 months of follow-up (primary endpoint). [3] Further assuming a 20% loss to follow-up, the final sample size estimate was 22 patients per arm. Statistical analysis was carried out using SPSS^®^ version 15.0 for Windows^®^ (SPSS, Chicago, Illinois, USA). Comparisons between groups were performed using the χ^2^ test or Fisher exact test for categorical variables, and the Mann-Whitney U test for continuous data. The paired t- test was used for the comparison of preoperative and postoperative biochemical data and data are presented as mean ± standard deviation. For comparing longitudinal variables according to reconstruction methods, repeated measured analysis of variance (RMANOVA) and linear mixed model was used and *P*-value was from interaction between time and reconstruction methods. When *P* -value of Mauchly’s test of sphericity was less than .05, Greenhous-Geisser correction was applied. *P-*values < .05 were considered statistically significant.

## Results

### Patient demographics and perioperative outcomes

**Fig 1:**
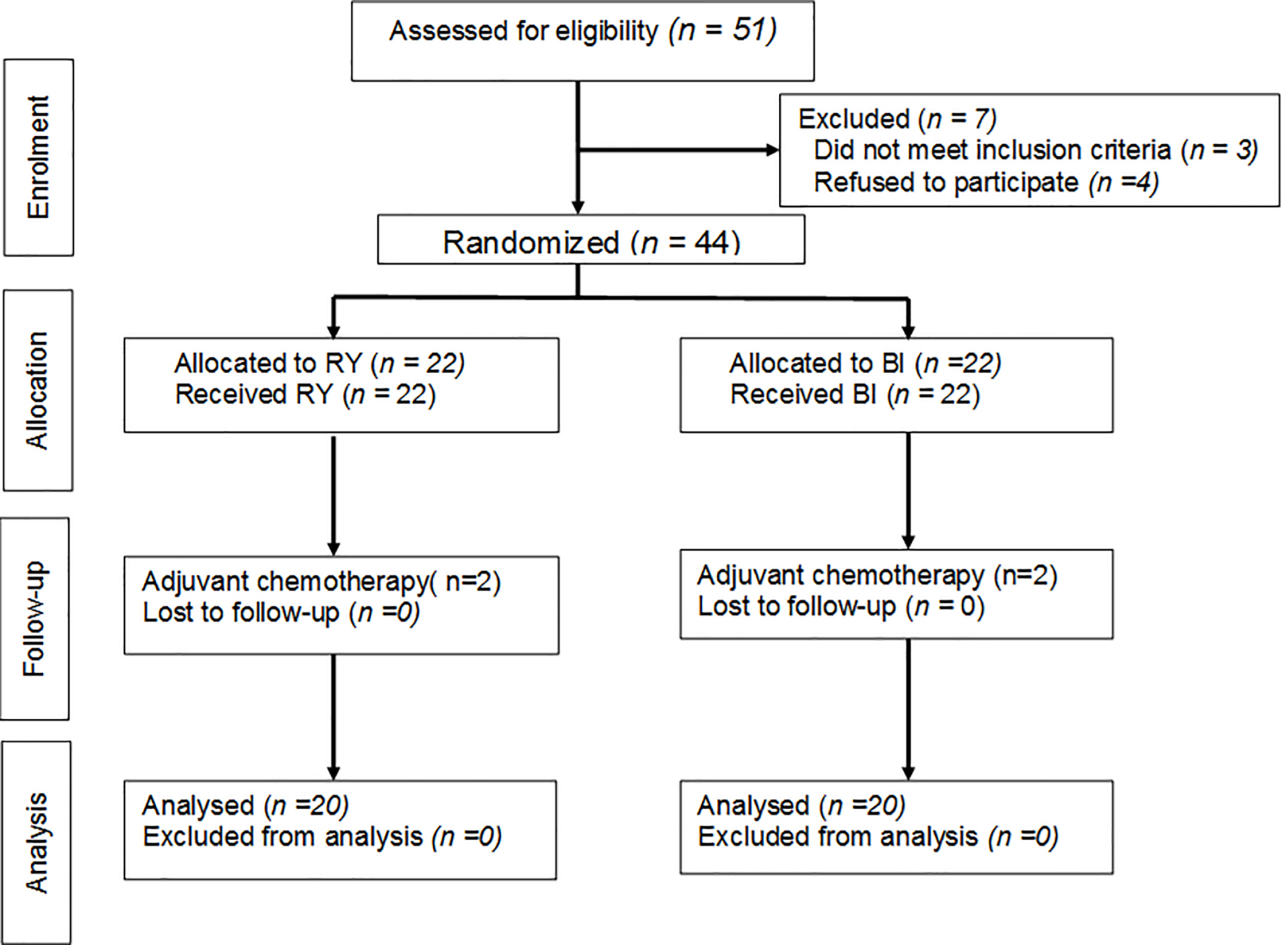
CONSORT diagram for trial.

Fifty one patients were initially screened for this study, and 3 patients did not meet the inclusion criteria and 4 patients refused to participate this study. Forty-four patients were randomized and then 2 patients (9.1%) in the RY group and 2 (9.1%) in the BI group were excluded due to postoperative chemotherapy. Finally, 40 patients successfully completed the study (Fig 1). Preoperative characteristics and operative factors were comparable between the RY and the BI groups, as shown in Table 1.

**Table 1:** Background and operative data.

Operation type	Roux-en-Y (n = 20) N(%)	Billroth I (n = 20) N(%)
**Age (years)**	62.8 ± 9.3	62.4 ± 8.9
**Sex**		
**Male**	10 (50.0)	16 (80.0)
**Female**	10 (50.0)	4 (20.0)
**Height (cm)**	160.3 ± 7.7	164.7 ± 7.7
**Weight (kg)**	68.3 ± 10.3	67.4 ± 13.1
**Body mass index (kg/m**^**2**^**)**	26.6 ± 4.3	24.7 ± 3.6
** Healty range (18.5–22.9)**	1 (5.0)	7 (35.0)
** Overweight (23–27.4)**	14 (70.0)	9 (45.0)
** Obese (27.5&above)**	5 (25.0)	4 (20.0)
**DM duration**		
** > 5 years**	10 (50.0)	10 (50.0)
** ≤ 5 years**	10 (50.0)	10 (50.0)
**DM medication**		
**Oral agents**	18 (90.0)	17 (85.0)
**Insulin only**	1 (5.0)	1 (5.0)
**Both**	1 (5.0)	2 (10.0)
**Fasting glucose**	134.1 ± 40.2	136.2 ± 37.4
**HbA1c (%)**	7.2 ± 0.9	7.6 ± 1.2
**HTN**	11 (55.0)	9 (45.0)
**pT stage**		
** T1**	19 (95.0)	17 (85.0)
** T2**	1 (5.0)	2 (10.0)
** T3**	0	1 (5.0)
**Hospital stay (days)**	7.1 ± 1.1	6.7 ± 1.0
**range**	6~11	5~9
**Lymph node dissection**		
** D1+**	8 (40.0)	9 (45.0)
** D2**	12 (60.0)	11 (55.0)
**Retrieved LN number**	29.6 ± 10.7	29.7 ± 11.6
**Blood loss during surgery (g)**	69.9 ± 53.4	93.1 ± 54.5
**Postoperative complication (all grade II)**	2 (10.0)	2 (10.0)
	1 atelectasis	1 atelectasis
	1 intraabdominal bleeding	1 intraabdominal complicated fluid

Data are mean ± standard deviation or numbers (percentages)

DM, diabetes mellitus; HTN, hypertension; LN, lymph node

Even though the preoperative tumor stage was early gastric cancer, 4 patients (10%, one patient in RY group and three patients in BI group) showed advanced gastric cancer in the final pathological report and none of the patients had lymph node metastasis. Because diabetes duration was a stratification factor for patient enrollment, the distribution of diabetes duration was same and preoperative fasting glucose and HbA1c levels were similar in both groups. Fifty percent of patients (11 and 9 patients in RY and BI group, respectively) in this study had accompanying hypertension. The extent of lymph node dissection, intraoperative bleeding and length of hospital stay were similar between the two groups. Postoperative complications occurred in two patients (10%) in each group, and there were no cases of grade III or IV complications, no cases of anastomotic or stump leaks and no reoperation. None of patients were suffered from significant gastrointestinal distress symptoms including dumping syndrome.

### Diabetes control after surgery

Patients were divided into three groups based on their diabetes status: stationary, improved and remission (Table 2). Among the 40 patients, 16 patients (40.0%) improved and only 1 patient (2.5%) went into remission at 12 months after surgery. In the RY group, 12 (60.0%) of the patients had improved and one of the patients stopped their medication at 12 months after surgery. In the BI group, one patient (5%) went into remission and 4 patients (20%) improved at 12 months after surgery, and one patient (5%) stopped DM medication at 12 months. The frequency of stationary vs. improved/remission in RY and BI at 12 months after surgery was significantly different (12 and 5 patients in RY and BI group, respectively, P = 0.025). The frequency of improved/remission patients was gradually increased in the RY group; 4 (20%), 6 (30%), 9 (45%), and 12 (60%) of patients at 3, 6, 9, 12 months after surgery. Before surgery, five among forty patients (12.5%, two and three patients in RY and BI group, respectively) were treated DM by insulin. Among those five patients, one patient in each group stopped insulin treatment 12 months after surgery.

**Table 2:** Diabetic control status and medication changes after surgery.

	3 months			6 months			9 months			12 months^†^		
	RY	BI	*P*-value*^‡^	RY	BI	*P*-value*^‡^	RY	BI	*P*-value*°	RY	BI	*P*-value*°
**Stationary**	16 (80)	17 (85)	>0.999	14 (70)	17 (85)	0.451	11 (55)	15 (75)	0.185	8 (40)	15 (75)	**0.025**
**Improved**	3 (15)	2 (10)		6 (30)	3 (15)		9 (45)	5 (25)		12 (60)	4 (20)	
**Remission**	1 (5)	1 (5)		0	0		0	0		0	1 (5)	
**Medication stop**	0	2 (10)		0	1 (5)		1 (5)	0		1 (5)	1 (5)	

Stationary: No change of medication, or patients except improved and remission criteria

Improved: Reduced medication and FBS <126 mg/dL and HbA1c < 6.5%

Remission: No medication and FBS <126 mg/dL and HbA1c < 6.0%

DM, diabetes mellitus; RY, subtotal gastrectomy, Roux-en-Y gastrojejunostomy; BI, subtotal gastrectomy, gastroduodenostomy

^†^ Primary end point of this study

*for Stationary vs. Improved/Remission

^‡^ Fisher’s exact test

° Chi-square test

### Changes in biochemical data after surgery

**Fig 2:**
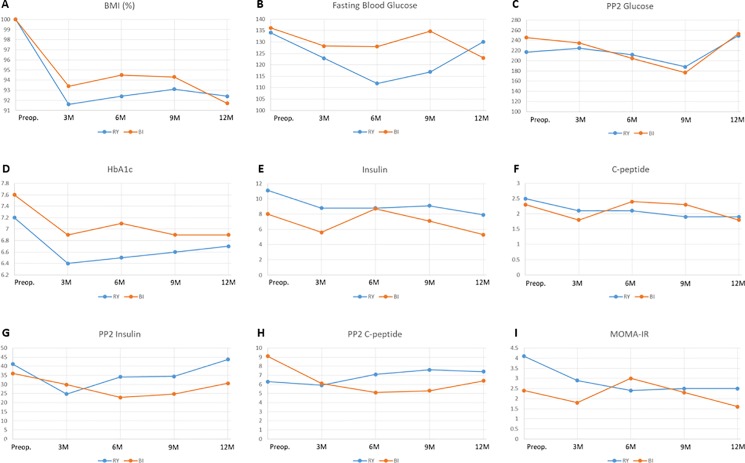
Serial changes in biochemical data reflecting glucose intolerance after surgery. RY, subtotal gastrectomy, Roux-en-Y gastrojejunostomy; BI, subtotal gastrectomy, gastroduodenostomy, BMI, body mass index; PP2, postprandial 2 hour; HOMA-IR, homeostasis model assessment-estimated insulin resistance.

Serial changes in the preoperative, postoperative 3, 6, 9, and 12 month BMI, FBS, PP2 glucose, HbA1c, insulin, C-peptide, and HOMA-IR are shown in Fig 2. BMI rapidly decreased during 3 months after surgery and it was maintained until 12 months (Fig 2A). Although the 12-month postoperative BMI value was significantly decreased to 92.4% and 91.7% of the preoperative value in the RY and BI groups, respectively (P<0.001), there was no significant difference between the RY group and the BI group at the same follow-up point.

FBS levels were more decreased in the RY group than in the BI group during 6 months after surgery, but the FBS level recovered at the 12 month follow-up in both groups (Fig 2B). The 12-month postoperative PP2 glucose level showed no significant difference according to the operation type (Fig 2C). In addition, the preoperative and postoperative 12 month values were similar in both groups.

HbA1c levels were rapidly decreased at 3 months after surgery, and then they rose slowly or were maintained up to 12 months after surgery in both groups (Fig 2D). Although the postoperative 12-month HbA1c levels were significantly decreased compared to the preoperative values in both groups (P = 0.025 in the RY group, 0.014 in the BI group), they showed no difference between the RY and BI groups (P = 0.265).

Fasting insulin and C-peptide levels showed a slow and continuous decrease until 12 months in the RY group, but they showed fluctuation during follow-up in the BI group (Fig 2E and 2F). The postoperative 12-month fasting C-peptide levels were significantly decreased compared to the preoperative values in the RY (P = 0.004) and BI groups (P = 0.002). The postprandial 2-hour insulin and C-peptide levels showed no significant difference in the preoperative and postoperative 12-month evaluation (Fig 2G and 2H), and they showed no difference according to the operation type at 12 months after surgery. HOMA-IR levels were consistently improved at 3 and 6 months, and they were maintained at 9 and 12 months after surgery in the RY group and the levels at 12 months after surgery were significantly lower (P = 0.030) than the preoperative levels (Fig 2I). In the BI group, HOMA-IR levels fluctuated according to the follow-up point, but the levels at 12 months after surgery were significantly lower than the preoperative levels (P = 0.039). As shown in S1 Table, BMI, HbA1c, insulin, C-peptide, and HOMA-IR changed significantly over time in RY and BI groups but their changing patterns were similar in both groups.

### Gut hormone analysis

**Fig 3:**
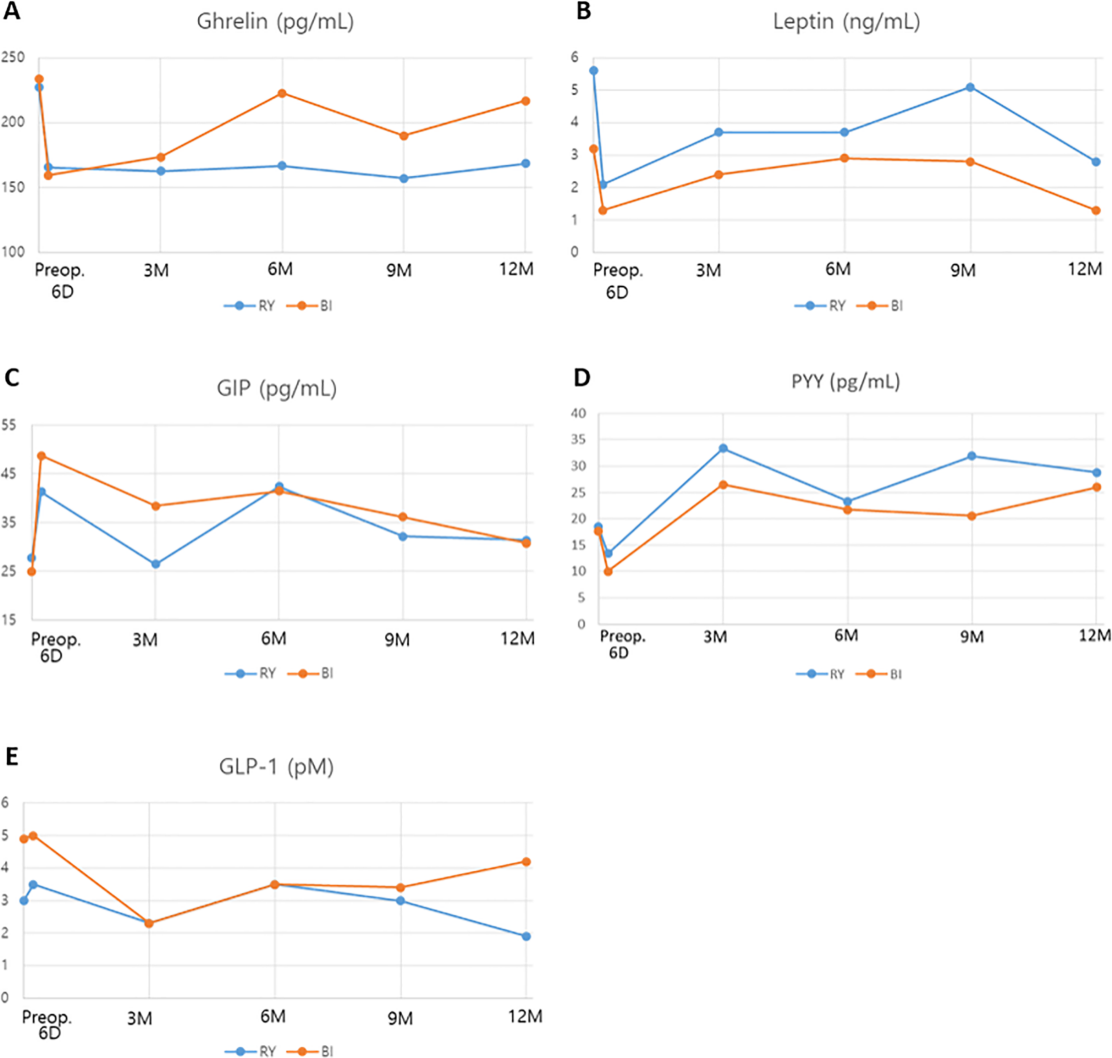
Gut hormone changes after surgery by reconstruction types. RY, subtotal gastrectomy with Roux-en-Y gastrojejunostomy; BI, subtotal gastrectomy with gastroduodenostomy; GLP-1, glucagon-like peptide-1; GIP, glucose-dependent insulinotropic polypeptide; PYY, Peptide YY.

We checked the levels of acylated ghrelin, leptin, GLP-1, GIP, and PYY in the fasting state (Fig 3). Ghrelin level was significantly decreased at 6 days after surgery in both groups (Fig 3A). After that, ghrelin level was increased up to the preoperative value in the BI group, but the decreased levels were maintained until 12 months after surgery in the RY group. The preoperative value and the postoperative 12- month value showed a significant difference in the RY group (P = 0.003), but not in the BI group (P = 0.449). Leptin levels were rapidly decreased in both RY and BI groups at the postoperative day 6, and then they increased up to 9 months and decreased again at 12 months after surgery (Fig 3B). These patterns were similar in both groups and the postoperative 12-month values were significantly lower than the preoperative values in both groups (P<0.001). GIP levels showed a rapid increase at postoperative day 6, but after that, they fluctuated at each follow-up point (Fig 3C). Finally, there was no significant difference between the preoperative and postoperative 12-month values of GIP. PYY levels were decreased at postoperative day 6, but after that, they increased significantly and high values were maintained up to 12 months after surgery in both groups (P<0.001 in the RY group, P = 0.021 in the BI group, Fig 3D). GLP-1 did not show any significant difference between the preoperative and postoperative values in both groups (Fig 3E). As shown in S2 Table, preoperative and postoperative 12 month values of ghrelin, leptin, and PYY were significantly different, but their changing patterns by time progress were not statistically different in RY and BI except ghrelin (*p* = 0.017).

### Factors for diabetes control at 12 months after surgery

We compared the improved and remission patients with those who were stationary to identify the predictive factors for diabetes control. Age, sex, BMI, reconstruction type, diabetes duration, preoperative blood glucose, HbA1c, insulin, C-peptide, HOMA-IR, ghrelin, leptin, GIP, PYY, and GLP-1 were analyzed (S3 Table). Improved/remission group showed higher frequency of female and RY reconstruction, lower preoperative FBS, PP2 glucose, HbA1c, ghrelin, and GIP than stationary group. Preoperative leptin level was higher in the improved/remission group than in the stationary group. However, we could not find out independent predictive factors for diabetic control at 12 months after surgery in multivariate logistic regression (data not shown). When it was adjusted by sex, RY was still related to better diabetic control despite it was not statistically significant (P = 0.089).

### Serial changes of gut hormones by the DM improvement status

We checked each hormonal changing pattern according to DM improvement status (S4 Table). Ghrelin, leptin, GIP and PYY level changed significantly in process of time but its changing pattern was not different between the improved/remission group and stationary group. The preoperative ghrelin level was significantly lower in improved/remission group than stationary group and it was decreased after surgery and maintained until 12 months with little fluctuation. Leptin, GIP, PYY levels also changed significantly in time process, but their changing patterns were not different between the improved/remission and stationary group.

## Discussion

As we know, this is the first randomized controlled trial of measuring diabetic improvement status and gut hormonal changes after gastric cancer surgery according to the reconstruction methods. In this randomized controlled trial of BI vs. RY in distal gastric adenocarcinoma, RY reconstruction showed a significantly higher incidence of diabetes improvement and a more durable reduction in insulin resistance compared to BI. During the 12-month follow-up, gut hormones including ghrelin, leptin, GIP, and PYY showed significant changes but relatively similar changing patterns in both groups except ghrelin; the levels of ghrelin was decreased in both group but the amount of change was larget in RY group than BI reconstruction. Gastric cancer surgeons have paid attention to the anti-diabetic effect of gastric cancer surgery, because there is similarity between bariatric surgery and gastric cancer surgery and improvement of T2DM after bariatric surgery have been reported even for patients with less obese (BMI ≤ 30 kg/m2) regardless of the amount of weight loss.[7, 8] Several studies showed that gastric cancer patients with T2DM experienced DM improvement after gastric cancer surgery in connection with weight loss, gastric resection and reconstruction. [2, 9–11] While some retrospective studies reported that the reconstruction methods including duodenal bypass such as BII and RY were more effective in producing DM improvement than BI, [9, 11, 12] our retrospective study of prospectively collected data showed that the pattern of biochemical data was similar in BI and BII reconstruction. [2] Because we presumed that incomplete bypass of the short segment of proximal small bowel (BII) did not provide significant additional benefits in terms of glucose metabolism, it can be hypothesized that RY reconstruction bypassing the duodenum and the proximal jejunum would have a better effect on diabetes control than BI reconstruction. [3]

As shown in Fig 2, fasting and postprandial serum glucose levels and HbA1c were decreased at 3 to 6 months after surgery, but then they increased until 12 months. Although the postoperative 12-month HbA1c level was significantly lower than the preoperative level, the preoperative and postoperative 12-month fasting and postprandial glucose levels showed no significant difference between both groups. These findings may be related to the increased calorie intake, weight regain and general condition recovery that occur by 3 months after gastrectomy. However, fasting serum insulin, C-peptide, and HOMA-IR levels dropped at 12 months after surgery in both groups. Interestingly, the levels constantly decreased in the RY group without any fluctuation, but they showed fluctuation at 6 months in the BI group, which suggests that RY is more effective for achieving a reduction in insulin resistance than BI.

We checked the levels of several gut hormones to evaluate the hormonal changes after surgery according to the reconstruction method and the degree of DM improvement. In our study, ghrelin showed interesting changes. Ghrelin is mainly produced in the stomach and the proximal small intestine, which contributes to the positive energy balance and weight gain by enhancing the appetite and adiposity. [13] Ghrelin suppression was reported to reduce fasting glucose and insulin levels, and to improve glucose tolerance. [1, 14] In our study, ghrelin level was decreased significantly at 6 days after gastrectomy in both BI and RY groups, but the decreased level was continuously maintained in the RY group, while it fluctuated and increased in the BI group and this difference was statistically significant. This pattern was similar to the changing patterns of insulin, C-peptide, and HOMA-IR, which suggests that RY has a more durable influence on ghrelin secretion and improvement of insulin resistance than BI. When we divided patients into two groups according to diabetic improvement status, preoperative ghrelin level was significantly lower and it decreased continuously with little fluctuation after surgery in the improved/remission group compared to the stationary group. Preoperative low ghrelin level and its maintenance after RY bypass appear to have an important role for diabetic control.

The postoperative 12- month leptin level was significantly lower than the preoperative level in both BI and RY groups. Considering that the leptin level corresponds closely with total body fat content, weight loss that develops after gastrectomy is connected with the decreased leptin level. GLP-1 is secreted in the terminal ileum and the postprandial GLP-1 level is increased after bariatric surgery. [1] In our study, we did not check the postprandial GLP-1 level but only the fasting GLP-1 level, which did not show a significant difference between the preoperative and postoperative values in both RY and BI groups. PYY is also secreted from the distal gastrointestinal tract, which increases in response to food intake and reach a peak at 1–2 hours after food intake, contributing to postprandial satiety and body weight loss. [14] It has been reported that PYY levels were markedly increased after bariatric surgery. [14, 15] In our study, PYY levels were increased significantly after surgery in both BI and RY groups, and it was significantly higher in the RY than in the BI at postoperative 9 months (P = 0.012) even though there was no difference at other follow-up points. The postoperative changing pattern of PYY was similar in DM improvement/remission group and stationary group. GIP is mainly secreted in the duodenum and upper jejunum, and its secretion is induced by food intake. As an incretin hormone, GIP stimulates insulin secretion in β-cells, inhibits gastric acid secretion, suppress lipolysis in adipose tissue, induces appetite and reduces energy expenditure in the brain. [16] In our study, GIP levels were rapidly increased at 6 days after surgery, but there was no difference between the preoperative level and the postoperative 12-month level and between the BI and RY groups. Considering that the postoperative GIP levels were higher than the preoperative levels, GIP secretion seems to be stimulated by gastric cancer surgery. As shown in S3 and S4 Tables, the preoperative lower GIP level was related to a better outcome of DM and the lower baseline GIP levels may indicate that the islet cell function is less impaired.

This study has several limitations. The small sample size of this study lead underpowered results with large variance in some variables and made it difficult to interpret the changes of hormones such as ghrelin, leptin, GLP-1, GIP, and PYY which were similar in the RY and BI groups. Also, statistical adjustment for multiplicity was not conducted. Thus it was difficult to clarify the difference in hormone secretion according to the diabetic improvement status and the mechanism of diabetic control, consequently the results need to be interpreted with caution of potential misleading. In addition, there were limitations to suitable interpretation due to the lack of postprandial hormonal levels, and short follow-up duration. Diet habit of each patient could affect to the result but it was neglected in this study, even all of the patients received nutrition education and randomization would minimize its’ influence. The duration of DM which was the only stratification factor was decided the time when the patient was diagnosed the DM that could be a lead time bias. To evaluate the exact mechanism of improvement of DM according to reconstruction methods in gastric cancer patients with T2DM with overcoming those limitations, we are going to conduct next clinical trial that includes a larger number of patients and performs the postprandial hormonal assay with longer follow-up periods.

In our study, the RY group showed a higher incidence of diabetes improvement (60%), which was a more durable improvement in insulin resistance, than the BI group (25%). Moreover, biochemical markers reflecting insulin resistance such as HbA1c, insulin, C-peptide, and HOMA-IR showed more durable improvement in RY compared to BI. These results suggest that RY reconstruction can be a favorable option for gastric cancer patients with DM and the modification of bypass length of the proximal small bowel would provide a better outcome for diabetes control. In other studies, RY reconstruction or longer modification of the RY limb in gastric cancer surgery showed a better outcome in diabetes improvement than the other reconstruction methods. [17, 18] Even though female was also related to higher incidence of diabetic improvement, RY reconstruction showed better effect on diabetic control than female sex after factor adjustment in multivariate logistic regression. Because of small number of patients, it was difficult to find out the independent predictive factors for diabetic control.

In conclusion, gastric cancer surgery led to a significant improvement in T2DM during the first year after surgery and RY reconstruction had a better impact on diabetes control than BI. After gastric cancer surgery, ghrelin and leptin levels were decreased and PYY and GIP levels were increased, which may have a role in improving insulin resistance. However, the impact of each reconstruction type on gut hormone secretion remains to be determined.

## Supporting information

S1 Fig Diagrams of each reconstruction type. A) Billroth I, B) Billroth II, C) Roux-en-Y gastrojejunostomy. S, stomach; D, duodenum; J, jejunum.
(TIF)

S1 Table Changes in biochemical data after surgery according to the reconstruction method. (DOCX)

S2 Table Hormonal changes after surgery. (DOCX)

S3 Table Preoperative clinical and biochemical factors in association with the diabetic control at 12 months after surgery. (DOCX)

S4 Table Hormonal changes according to the DM improvement status. (DOCX)

S1 File CONSORT checklist. Checklist of this manuscript according to CONSORT guideline.
(DOC)

S2 File Translated protocol. The translated protocol of this study which was approved by Institutional Review Board by English.
(DOCX)

S3 File Original protocol in Korean. The original protocol of this study which was approved by Institutional Review Board.
(DOC)
