# Supplementary figures and images for: A randomized controlled trial of Roux-en-Y gastrojejunostomy vs. gastroduodenostomy with respect to the improvement of type 2 diabetes mellitus after distal gastrectomy in gastric cancer patients
Source: PLoS One. 2017 Dec 7;12(12):e0188904. doi: 10.1371/journal.pone.0188904 (PMC5720795; doi:10.1371/journal.pone.0188904)

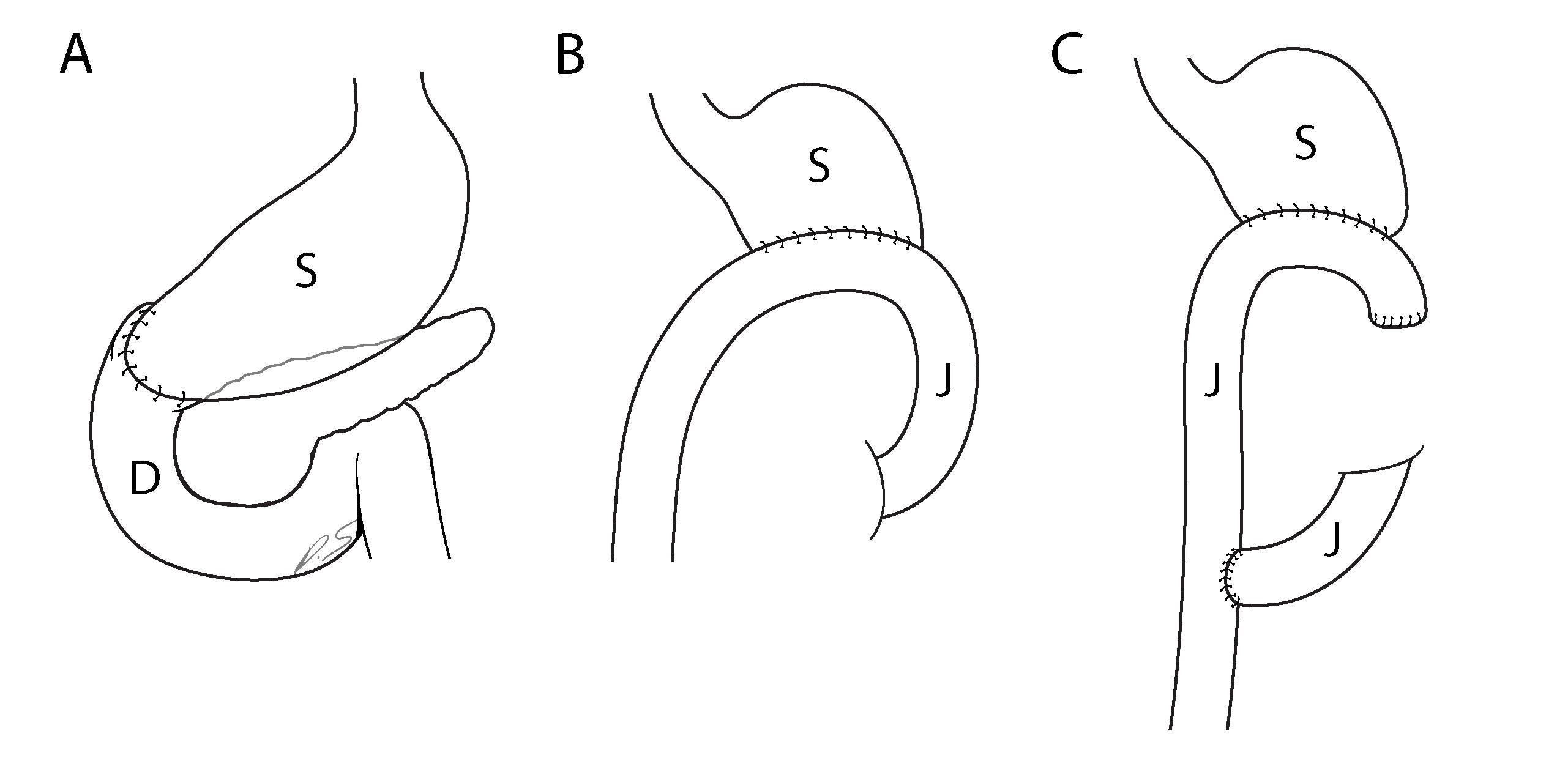

Supplement: S1 Fig — A) Billroth I, B) Billroth II, C) Roux-en-Y gastrojejunostomy. S, stomach; D, duodenum; J, jejunum. (TIF) [file pone.0188904.s001.tif]
