# Supplementary material for: A randomized controlled trial of Roux-en-Y gastrojejunostomy vs. gastroduodenostomy with respect to the improvement of type 2 diabetes mellitus after distal gastrectomy in gastric cancer patients
Source: PLoS One. 2017 Dec 7;12(12):e0188904. doi: 10.1371/journal.pone.0188904 (PMC5720795; doi:10.1371/journal.pone.0188904)
Supplement: S1 Table — (DOCX) [file pone.0188904.s002.docx]

**S1 Table. Changes in biochemical data after surgery according to the reconstruction method**

|  |  | **Preoperative** | **3 moths** | **6 months** | **9 months** | **12 months** | ***P*-value**^*^  **(Pre- 12M)** | ***P*-value**^†^ |
| --- | --- | --- | --- | --- | --- | --- | --- | --- |
| **BMI (%)** | **RY** | 100 | 91.6 ± 4.9 | 92.4 ± 5.4 | 93.1 ± 6.3 | 92.4 ± 6.3 | **<0.001** | 0.195° |
|  | **BI** | 100 | 93.4 ± 5.2 | 94.5 ± 5.2 | 94.3 ± 4.9 | 91.7 ± 5.9 | **<0.001** |  |
| **Fasting glucose (mg/dL)** | **RY** | 134.1 ± 40.2 | 122.9 ± 28.8 | 111.8 ± 20.1 | 116.9 ± 33.8 | 130.1 ± 35.7 | 0.684 | 0.155° |
|  | **BI** | 136.2 ± 37.4 | 128.2 ± 25.5 | 128.0 ± 35.1 | 134.7 ± 32.7 | 123.0 ± 28.7 | 0.159 |  |
| **PP2 glucose**  **(mg/dL)** | **RY** | 217.0 ±79.3 | 224.7 ± 80.9 | 211.8 ± 76.5 | 188.1 ± 75.9 | 249.2 ± 76.5 | 0.191 | 0.703 |
|  | **BI** | 245.6 ± 67.7 | 234.9 ± 66.0 | 204.5 ± 60.4 | 176.7 ± 86.4 | 252.9 ± 54.0 | 0.670 |  |
| **HbA1c (%)** | **RY** | 7.2 ± 0.9 | 6.4 ± 0.5 | 6.5 ± 0.6 | 6.6 ± 0.6 | 6.7 ± 0.6 | **0.025** | 0.265° |
|  | **BI** | 7.6 ± 1.2 | 6.9 ± 0.8 | 7.1 ± 0.8 | 6.9 ± 0.6 | 6.9 ± 0.6 | **0.014** |  |
| **Insulin (μIU/mL)** | **RY** | 11.1 ± 13.7 | 8.8 ± 6.3 | 8.8 ± 5.7 | 9.1 ± 8.5 | 7.9 ± 4.6 | 0.266 | 0.447° |
|  | **BI** | 8.0 ± 4.8 | 5.6 ± 3.7 | 8.7 ± 6.5 | 7.1 ± 2.6 | 5.3 ± 2.4 | **0.032** |  |
| **PP2 Insulin (μIU/mL)** | **RY** | 41.2 ± 39.3 | 24.8 ± 24.4 | 34.2 ± 26.3 | 34.4 ± 29.8 | 43.7 ± 53.3 | 0.741 | 0.674° |
|  | **BI** | 36.1 ± 20.1 | 29.9 ± 32.1 | 22.9 ± 14.9 | 24.8 ± 16.6 | 30.6 ± 25.7 | 0.447 |  |
| **C-peptide (ng/mL)** | **RY** | 2.5 ± 0.9 | 2.1 ± 0.8 | 2.1 ± 0.7 | 1.9 ± 0.7 | 1.9 ± 0.6 | **0.004** | 0.098° |
|  | **BI** | 2.3 ± 0.9 | 1.8 ± 0.7 | 2.4 ± 1.4 | 2.3 ± 1.2 | 1.8 ± 0.6 | **0.002** |  |
| **PP2**  **C-peptide (ng/mL)** | **RY** | 6.3 ± 2.7 | 5.9 ±3.1 | 7.1 ± 3.2 | 7.6 ± 4.6 | 7.4 ± 3.9 | 0.305 | 0.089° |
|  | **BI** | 9.1 ± 11.9 | 6.1 ± 3.4 | 5.1 ± 2.3 | 5.3 ± 2.3 | 6.4 ± 2.9 | 0.291 |  |
| **HOMA-IR** | **RY** | 4.1 ± 7.0 | 2.9 ± 2.5 | 2.4 ± 1.5 | 2.5 ± 2.4 | 2.5 ± 1.5 | **0.030** | 0.241° |
|  | **BI** | 2.4 ± 1.6 | 1.8 ± 1.2 | 3.0 ± 2.7 | 2.3 ± 0.9 | 1.6 ± 0.7 | **0.039** |  |

^*^Paired t-test, mean ± standard deviation, preoperative value and postoperative 12 month value was compared.

^†^Interaction between time and reconstruction method for Repeated Measured Analysis of Variance (RMANOVA)

°Greenhous-Geisser correction

BMI (%), percentage of BMI at each follow up compared to preoperative BMI

BI, subtotal gastrectomy, gastroduodenostomy; RY, subtotal gastrectomy, Roux-en-Y gastrojejunostomy; ; HOMA-IR, homeostasis model assessment-estimated insulin resistance; PP2, postprandial 2 hour
