# Supplementary material for: A randomized controlled trial of Roux-en-Y gastrojejunostomy vs. gastroduodenostomy with respect to the improvement of type 2 diabetes mellitus after distal gastrectomy in gastric cancer patients
Source: PLoS One. 2017 Dec 7;12(12):e0188904. doi: 10.1371/journal.pone.0188904 (PMC5720795; doi:10.1371/journal.pone.0188904)
Supplement: S2 Table — (DOCX) [file pone.0188904.s003.docx]

**S2 Table. Hormonal changes after surgery**

|  |  | **Preop** | **6D** | **3M** | **6M** | **9M** | **12M** | ***P*-value**^*^  **(Pre- 12M)** | ***P*-value**^†^ |
| --- | --- | --- | --- | --- | --- | --- | --- | --- | --- |
| **Ghrelin**  **(pg/mL)** | **RY** | 227.0±136.4 | 165.6±94.1 | 162.6±122.2 | 166.8±88.4 | 157.0±73.0 | 168.5±108.4 | **0.003** | **0.017** |
|  | **BI** | 233.9±152.5 | 159.4±83.7 | 173.6±127.0 | 222.7±124.1 | 190.0±86.2 | 216.8±99.4 | 0.449 |  |
| **Leptin**  **(ng/mL)** | **RY** | 5.6±3.9 | 2.1±1.8 | 3.7±3.5 | 3.7±2.6 | 5.1±4.0 | 2.8±2.1 | **0.000** | 0.2923 |
|  | **BI** | 3.2±2.2 | 1.3±1.7 | 2.4±2.6 | 2.9±3.5 | 2.8±2.2 | 1.3±1.3 | **0.000** |  |
| **GLP-1**  **(pM)** | **RY** | 3.0±3.4 | 3.5±2.1 | 2.3±1.8 | 3.5±2.5 | 3.0±3.4 | 1.9±1.6 | 0.150 | 0.958 |
|  | **BI** | 4.9±8.3 | 5.0±7.5 | 2.3±2.7 | 3.5±2.9 | 3.4±2.8 | 4.2±11.0 | 0.817 |  |
| **GIP**  **(pg/mL)** | **RY** | 27.7±12.7 | 41.3±20.0 | 26.5±15.6 | 42.4±28.3 | 32.2±19.0 | 31.4±18.7 | 0.341 | 0.830 |
|  | **BI** | 24.9±12.4 | 48.7±23.5 | 38.4±21.0 | 41.5±27.1 | 36.2±22.9 | 30.8±21.5 | 0.217 |  |
| **PYY**  **(pg/mL)** | **RY** | 18.5±12.0 | 13.4±18.0 | 33.4±22.1 | 23.3±14.9 | 31.9±14.7 | 28.8±11.8 | **0.000** | 0.292 |
|  | **BI** | 17.7±11.3 | 10.0±13.5 | 26.5±13.6 | 21.7±12.4 | 20.6±11.8 | 26.0±14.8 | 0.021 |  |

^*^Paired t-test, mean ± standard deviation between preoperative level and postoperative 12 month level.

^†^Interaction between time and reconstruction method for Linear Mixed Model

RY, subtotal gastrectomy, Roux-en-Y gastrojejunostomy; BI, subtotal gastrectomy, gastroduodenostomy; HOMA-IR, homeostasis model assessment-estimated insulin resistance
