# Supplementary material for: A randomized controlled trial of Roux-en-Y gastrojejunostomy vs. gastroduodenostomy with respect to the improvement of type 2 diabetes mellitus after distal gastrectomy in gastric cancer patients
Source: PLoS One. 2017 Dec 7;12(12):e0188904. doi: 10.1371/journal.pone.0188904 (PMC5720795; doi:10.1371/journal.pone.0188904)
Supplement: S3 Table — (DOCX) [file pone.0188904.s004.docx]

**S3 Table. Preoperative clinical and biochemical factors in association with the diabetic control at 12 months after surgery**

|  | **Improved/Remission**  **(n=17)** | **Stationary**  **(n=23)** | ***P*-value**^*^ |
| --- | --- | --- | --- |
| **Age (years)** | 62.4 ± 9.8 | 62.7 ± 8.9 | 0.909 |
| **Sex, Female (%)** | 9 (52.9) | 5 (21.7) | **0.041^†^** |
| **BMI preop (kg/m^2^ )**  **12 months** | 26.7 ± 4.7  24.3±3.4 | 24.9 ± 3.4  23.1 ± 3.5 | 0.570  0.342 |
| **Reconstruction type**  **RY**  **BI** | 12 (70.6)  5 (29.4) | 8 (34.8)  15 (65.2) | **0.025^†^** |
| **DM duration** | 5.9 ± 5.7 | 7.1 ± 5.7 | 0.401 |
| **Fasting glucose (mg/dL)** | 121.7 ± 30.6 | 145.0 ± 41.1 | **0.045** |
| **PP2 glucose (mg/dL)** | 202.5 ± 62.2 | 252.6 ± 76.4 | 0.034 |
| **HbA1c (%)** | 7.0 ± 1.1 | 7.7 ± 0.9 | **0.008** |
| **Insulin (μIU/mL)** | 7.5 ± 3.6 | 11.0 ± 13.1 | 0.725 |
| **C-peptide (ng/mL)** | 2.2 ± 0.8 | 2.5 ± 1.0 | 0.290 |
| **HOMA-IR** | 2.2 ± 1.0 | 4.1 ± 6.6 | 0.401 |
| **Ghrelin** | 170.1 ± 66.0 | 275.1 ± 167.7 | **0.025** |
| **Leptin** | 5.9 ± 4.0 | 3.3 ± 2.2 | 0.048 |
| **GLP-1** | 2.4 ± 3.4 | 5.2 ±7.7 | 0.101 |
| **GIP** | 20.9 ± 11.0 | 30.2 ± 12.2 | **0.022** |
| **PYY** | 17.5 ± 12.9 | 18.4 ± 10.8 | 0.726 |

*Mann-Whitney U test, ^†^Chi-square,

Stationary: No change of medication, or patients except improved and remission criteria

Improved: Reduced medication and FBS <126 mg/dL and HbA1c < 6.5 %

Remission: No medication and FBS <126 mg/dL and HbA1c < 6.0 %

BMI, body mass index; DM, diabetes mellitus; RY, subtotal gastrectomy with Roux-en-Y gastrojejunostomy; BI, subtotal gastrectomy with gastroduodenostomy; FBS, fasting blood glucose; PP2, postprandial 2 hour; HOMA-IR, homeostasis model assessment-estimated insulin resistance; GLP-1, glucagon-like peptide-1; GIP, glucose-dependent insulinotropic polypeptide; PYY, Peptide YY
