# Supplementary material for: A randomized controlled trial of Roux-en-Y gastrojejunostomy vs. gastroduodenostomy with respect to the improvement of type 2 diabetes mellitus after distal gastrectomy in gastric cancer patients
Source: PLoS One. 2017 Dec 7;12(12):e0188904. doi: 10.1371/journal.pone.0188904 (PMC5720795; doi:10.1371/journal.pone.0188904)
Supplement: S4 Table — (DOCX) [file pone.0188904.s005.docx]

**S4 Table. Hormonal changes according to the DM improvement status**

|  | **Improved/Remission**  **(n=17)** | **Stationary**  **(n=23)** | ***P*-value**^*^ | ***P* -value**^†^ |
| --- | --- | --- | --- | --- |
| **Ghrelin** |  |  | <0.001 | 0.269 |
| **Preop** | 170.14±66.01 | 256.05±167.71 |  |  |
| **6D** | 143.28±80.08 | 174.74±94.24 |  |  |
| **3M** | 116.88±86.83 | 199.75±133.31 |  |  |
| **6M** | 152.89±74.18 | 220.81±123.49 |  |  |
| **9M** | 145.00±67.11 | 194.78±84.44 |  |  |
| **12M** | 145.17±77.06 | 219.83±107.13 |  |  |
| **Leptin** |  |  | <0.001° | 0.108° |
| **Preop** | 5.94±4.03 | 3.32±2.19 |  |  |
| **6D** | 2.02±1.76 | 1.37±1.72 |  |  |
| **3M** | 3.86±3.28 | 2.34±2.91 |  |  |
| **6M** | 4.38±3.50 | 2.49±2.63 |  |  |
| **9M** | 5.27±4.39 | 2.96±2.00 |  |  |
| **12M** | 2.76±2.30 | 1.44±1.17 |  |  |
| **GLP-1** |  |  | 0.584° | 0.690° |
| **Preop** | 2.39±3.38 | 5.15±7.69 |  |  |
| **6D** | 2.57±1.51 | 5.33±7.13 |  |  |
| **3M** | 1.90±1.69 | 2.75±2.55 |  |  |
| **6M** | 2.97±2.41 | 3.98±2.87 |  |  |
| **9M** | 2.89±3.08 | 3.56±2.68 |  |  |
| **12M** | 1.66±1.52 | 4.17±10.49 |  |  |
| **GIP** |  |  | <0.001° | 0.314° |
| **Preop** | 20.92±11.01 | 30.23±12.17 |  |  |
| **6D** | 40.64±19.14 | 47.88±34.96 |  |  |
| **3M** | 30.32±17.74 | 33.06±20.75 |  |  |
| **6M** | 42.97±30.03 | 37.40±18.80 |  |  |
| **9M** | 35.00±24.30 | 33.48±18.20 |  |  |
| **12M** | 32.04±21.02 | 30.50±20.00 |  |  |
| **PYY** |  |  | <0.001 | 0.843 |
| **Preop** | 17.48±12.98 | 18.43±10.80 |  |  |
| **6D** | 12.13±11.95 | 9.26±17.69 |  |  |
| **3M** | 32.67±19.58 | 27.75±18.57 |  |  |
| **6M** | 22.41±14.97 | 21.77±12.79 |  |  |
| **9M** | 28.22±14.24 | 23.51±13.25 |  |  |
| **12M** | 29.13±14.38 | 25.27±12.42 |  |  |

^*^Time for Repeated Measured Analysis of Variance (RMANOVA)

^†^Interaction between time and DM status for RMANOVA

°Greenhous-Geisser correction
