# Supplementary material for: A randomized controlled trial of Roux-en-Y gastrojejunostomy vs. gastroduodenostomy with respect to the improvement of type 2 diabetes mellitus after distal gastrectomy in gastric cancer patients
Source: PLoS One. 2017 Dec 7;12(12):e0188904. doi: 10.1371/journal.pone.0188904 (PMC5720795; doi:10.1371/journal.pone.0188904)
Supplement: S2 File — The translated protocol of this study which was approved by Institutional Review Board by English. (DOCX) [file pone.0188904.s007.docx]

**Proposal of clinical trial**

1. **Title:**

Improving Diabetes by Reconstruction Methods in Gastric Cancer Patients with Diabetes Mellitus

1. **Background**

**Surgical treatment of type II DM**

Recently, metabolic surgery has attracted attention as a new treatment method for a treatment of type 2 diabetes. Metabolic surgery was first introduced for the treatment of metabolic diseases such as hyperglycemia, as it was reported that the hyperglycemia of obese patients was improved as a result of surgery to reduce the body weight of obese patients.

Metabolic surgery is a surgical procedure that changes the pathways of food in gastrointestinal track. As laparoscopic surgery, which showed its’ benefit of less scar and early recovery from surgery, is introduced for metabolic surgery, attention to metabolic surgery have been accelerated. Adjustable gastric banding (AGB), Roux-en-Y gastric bypass (RYGB), biliopancreatic diversion (BPD) are the name of metabolic surgery, and RYGB and BPD are preferred procedure to control hyperglycemia.

RYGB is one of the common metabolic surgery procedures: After making small amount of gastric pouch, jejunum is resected 30-50 cm below treiz ligament and do RY anastomosis with 50-150 cm of Rou limb.


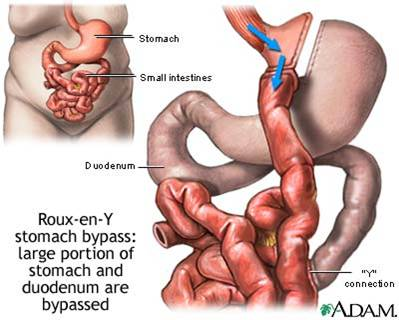


It was reported that eighty percent of hyperglycemia patients who underwent this surgery, experienced reduction of the amount of insulin hypoglycemic drug and insulin. Also this surgery was effective even patients with less than 35 kg/m^2^ of body mass index.

**The mechanism of improving diabetes by surgery**

Basically reducing oral intake and nutritional absorption in GI track is a mechanism of improving hyperglycemia by surgery. In addition, various hormones such as GLP-1, IGF-1, Leptin, CCK, Ghrelin, Neuropeptide Y, which is changed its level bypassing food can lead the improvement of impairment of insulin and hyperglycemia.

1. **The upper intestinal hypothesis**

There is a theory for the improvement of hyperglycemia for patients receiving metabolic surgery: if food does not pass through duodenum (food does not contact any upper GI mucosa), an unknown-hormone which related insulin resistance, is not secreted, consequently, it can improve hyperglycemia. A study showed that duodeno-jejunal bypass (DJB) could improve DM without notable weight loss or the amount of diet. Also another study showed that excluding duodenum for food passage led improving DM, but re-including duodenum led recurrence of DM. Another study which can valid this theory showed that endoluminal duodenal sleeve which prevent the contact between duodenal mucosa and food lead improving DM.


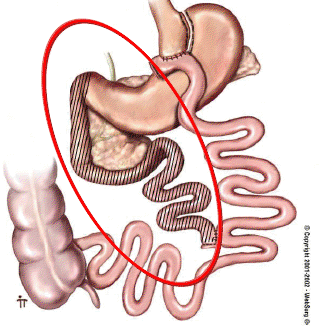


Proximal bowel hypothesis: When excluding proximal bowel including duodenum for food passage lead improving DM (Rubino et al. Ann Surg, 2008)

1. **The lower intestinal hypothesis**

RYGB and BPD procedure makes early contacting food to distal small bowel and it lead secretion of GLP-1, one of incretin peptide and this mechanism would be related to improving DM. GLP-1 induces glucose-dependent insulin secretion and inhibits glucagon secretion, and delays gastric empting. Also, it induced increasing mass of beta-cell in *in vivo* model. GLP-1 is stimulated by nutritional contacting at L-cell in ileum and colon and induces inhibition of appetite and reducing glucose level.


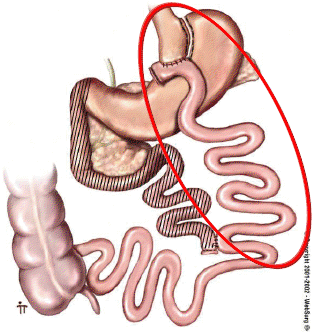


Distal bowel hypothesis: early contacting food to distal part of small bowel lead secretion of GLP-1 in L-cell and it stimulate the effect of glucagon. (Rubino et al. Ann Surg. 2008)

1. **The ghrelin hypothesis**

Ghrelin stimulate appetite and is a hormone related in impairment of DM control. It is increased just before diet and decreased after diet. Over 90% of Ghrelin is secreted at stomach and duodenum, and RYGB makes food bypassing stomach and duodenum, consequently this procedure would prevent secretion of ghrelin. It was reported that RYGB was related to low level of ghrelin. Because ghrelin inhibits adiponectin, one of hormone of insulin-sensitizing hormone, and block phosphatidylinositol-3-kinase signal in liver, reducing ghrelin level by RYGB would induce the control of DM.

**Surgical treatment for patients with gastric cancer and type II DM.**

Gastrectomy for gastric cancer leads weight loss of patient and sometimes the patient experience resolution of co-morbidity such as hypertension and DM. Therefore, modifying gastric cancer surgery with adaption of metabolic surgery could improve DM more efficiently. The main procedure for gastric cancer surgery is that resection of stomach including cancer and lymph node dissection. The extent of gastrectomy is decided not by the presence of DM but by location of tumor and surgeon’s preferences; gastroduodenostomy, gastrojejunostomy with loop type, and Roux-en-Y gastrojejunostomy are the common reconstruction type. The type of reconstruction is known that it is not related to the patients’ prognosis. Recent study reported that subtotal gastrectomy with Roux-en-Y reconstruction for gastric cancer was related improvement of DM; 57% of patients were not required any medication for DM at 1 year after gastrectomy. Thus, this study is going to compare the outcome of DM improvement after subtotal gastrectomy for patients with gastric cancer and DM between Roux-en-Y gastrojejunostomy and gastroduodenostomy.


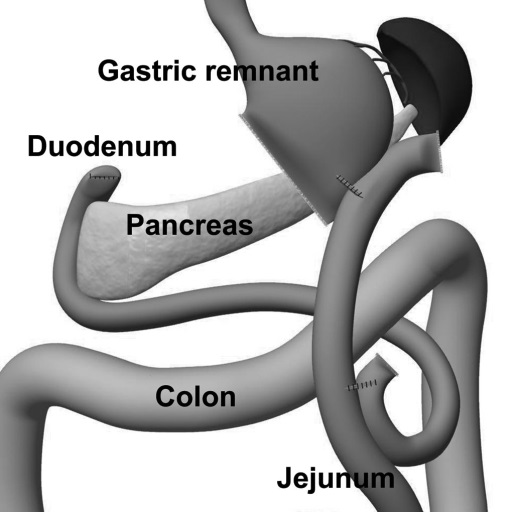

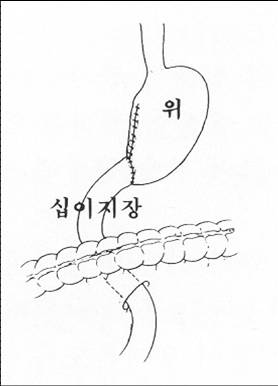


(Roux-en-Y gastrojejunostomy) (Gastroduodenostomy)

# Roux-en-Y gastrojejunostomy

Transecting jejunum was performed 25-30cm below treiz ligament after standard gastrectomy with lymphadenectomy. Side-to side jejuno-jejunostomy was performed 30-40cm below gastrojejunostomy.

# Gastroduodenostomy

Gastroduodenostomy was performed using circular staple and the common channel was repaired by linear stapler after standard gastrectomy with lymphadenectomy.

Until now, no randomized controlled trial has been conducted for comparing the efficacy of DM improvement according to reconstruction type for gastric cancer surgery.

1. **Purpose**
2. Comparing the effect of improvement of hyperglycemia according to reconstruction type (Roux-en-Y gastrojejunostomy vs. gastroduodenostomy) in patients with gastric cancer and DM
3. Evaluating the mechanism of DM improvement according to reconstruction type.
4. **Place and period of the study**

Place: Severance hospital, Yonsei University College of Medicine

Periods: 2011.05.01~2014.04.30 (36 months)

1. **Selection criteria**

Inclusion criteria

1. Plan to underwent surgery for early gastric cancer, and in treatment for diagnosed DM and/or newly diagnosed DM in pre-operative check up
2. Location of tumor is lower part of stomach (possible cases of distal gastrectomy)
3. Age between 20-80
4. Understand the study and informed consent patients

Exclusion criteria

1. Patients who were treated by systemic inflammatory disease or with other malignancy
2. Uncontrolled co-morbidity
3. Vulnerable subjects (minor, pregnant women, lack of ability of recognition)
4. Severe complication after gastrectomy
5. **Sample size**

Retrospective study from our institution showed that proportion of patients who experience complete remission of DM, which was defined as no more necessity drug to control DM, was around 15% after gastroduodenostomy. It was reported that around 57% of patients who underwent Roux-en-Y gastrojejunostomy for gastric cancer with DM experienced remission of DM. Thus sample size for each group will be 18 for 0.05 of alpha and 0.20 of beta, and estimating 10% of drop-out rate, total 40 patients will be required.

# **Two Independent Proportions (Null Case) Power Analysis**

**Numeric Results of Tests Based on the Difference: P1 - P2**

**H0: P1-P2=0. H1: P1-P2=D1<>0. Test Statistic: Likelihood Ratio test**

**Sample Sample Prop|H1 Prop**

**Size Size Grp 1 or Grp 2 or Diff Diff**

**Grp 1 Grp 2 Trtmnt Control if H0 if H1 Target Actual**

**Power N1 N2 P1 P2 D0 D1 Alpha Alpha Beta**

0.8091 18 18 0.5700 0.1500 0.0000 0.4200 0.0500 0.0764 0.1909

Note: exact results based on the binomial were only calculated when both N1 and N2 were less than 100.

**References**

Chow, S.C.; Shao, J.; Wang, H. 2003. Sample Size Calculations in Clinical Research. Marcel Dekker. New York.

D'Agostino, R.B., Chase, W., Belanger, A. 1988.'The Appropriateness of Some Common Procedures for Testing the Equality of Two Independent Binomial Populations', The American Statistician, August 1988, Volume 42 Number 3, pages 198-202.

Fleiss, J. L., Levin, B., Paik, M.C. 2003. Statistical Methods for Rates and Proportions. Third Edition. John

Wiley & Sons. New York.

Lachin, John M. 2000. Biostatistical Methods. John Wiley & Sons. New York.

Machin, D., Campbell, M., Fayers, P., and Pinol, A. 1997. Sample Size Tables for Clinical Studies, 2nd

Edition. Blackwell Science. Malden, Mass.

**Report Definitions**

'Power' is the probability of rejecting a false null hypothesis. It should be close to one.

'N1 and N2' are the sizes of the samples drawn from the corresponding populations.

'P1' is the proportion for group one under H1. This is the treatment or experimental group.

'P2' is the proportion for group two. This is the standard, reference, or control group

'Target Alpha' is the probability of rejecting a true null hypothesis that was desired.

'Actual Alpha' is the value of alpha that is actually achieved.

'Beta' is the probability of accepting a false null hypothesis.

**Summary Statements**

Group sample sizes of 18 in group one and 18 in group two achieve 81% power to detect a difference between the group proportions of 0.4200. The proportion in group one (the treatment group) is assumed to be 0.1500 under the null hypothesis and 0.5700 under the alternative hypothesis. The proportion in group two (the control group) is 0.1500. The test statistic used is the two-sided Likelihood Ratio test. The significance level of the test was targeted at 0.0500. The significance level actually achieved by this design is 0.0764.

The formula for estimation was like as follow:


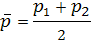


In our institution, around 50 patients underwent subtotal gastrectomy for stage I/II gastric cancer per year. Thus, 40 patients of enrollment will be possible within a year.

The duration of DM is a stratification factor for randimization and randon sequence is like as follow:

| **OBS** | **Duration of DM** | |
| --- | --- | --- |
|  | **>=5year** | **<5** |
| **1** | 1 | 2 |
| **2** | 2 | 1 |
| **3** | 2 | 2 |
| **4** | 1 | 1 |
| **5** | 2 | 2 |
| **6** | 1 | 1 |
| **7** | 1 | 2 |
| **8** | 2 | 1 |
| **9** | 1 | 2 |
| **10** | 2 | 1 |
| **11** | 1 | 2 |
| **12** | 2 | 1 |
| **13** | 2 | 2 |
| **14** | 1 | 1 |
| **15** | 1 | 2 |
| **16** | 2 | 1 |
| **17** | 1 | 1 |
| **18** | 2 | 2 |
| **19** | 2 | 1 |
| **20** | 1 | 2 |

1. **Study design and methods**

**<Study design>**

1. 0-1month: Get approval from IRB
2. 2-13 months: patients’ enrollment
3. 14-25 months: follow up with laboratory test
4. 26-36 months: analysis and report

**<Methods>**

- Selecting patients who satisfy inclusion and exclusion criteria
- Randomization (without blinding):
  - Experiment arm: Roux-en-Y gastrojejunostomy
  - Control arm: Gastroduodenostomy
- Laboratory follow up: preoperation, post-operative day#7, #3months, #12 months
- Weight, fasting sugar level, post prandial 2hr sugar level, HbA1c, C-peptide (both of fasting and post prandial 2hr), lipid profile, insulin (both of fasting and post prandial 2hr), glucagon, IGF-1, GLP-1, Neuropeptide Y, Ghrelin, Leptin
- HOMA scale: HI=plasma glucose(mmol.L) x insulin(UI/ml)/22.5
- Comparing the outcomes between Roux-en-Y gastrojejunostomy and Gastroduodenostomy

1. **Variables**

-height, weight

-blood pressure, pulse rate

-duration of DM, duration of DM treatment, drug for DM

-insulin or not and amount

-pathology, postoperative morbidity and mortality

-Weight, fasting sugar level, post prandial 2hr sugar level, HbA1c, C-peptide (both of fasting and post prandial 2hr), lipid profile, insulin (both of fasting and post prandial 2hr), glucagon, IGF-1, GLP-1, Neuropeptide Y, Ghrelin, Leptin

- Laboratory follow up: preoperation, post-operative day#7, #3months, #12 months

-questionaire for quality of life

***Evaluation of hormonal change**

(1) blood sampling and centrifuge for plasma

Sampling at follow up point

Keep 8hr of NPO, storage in EDTA tube, centrifuge at 4℃, 1600 x g for 15 minutes. Keep plasma at -70℃

(2) Radioimmunoassay (RIA)

<commercial RIA kits>

Ghrelin과 Leptin (Linco Research, St. Charles, Missouri)

GLP-1과 NPY (Interscience Institute, Inglewood, CA)

Glucagon, IGF-1 (Endocrine Sciences Inc., Calabasas, CA)

1. **Estimated side effects of this study**

No specific additional risk

Same as standard gastrectomy

1. **Trial drop-out and stop**

When participant withdraw his/her agreement

1. **Analysis and interpretation**

**Evaluation**

-Improvement of laboratory data about DM after surgery

-Decreasing of drug to control DM or remission of DM

**Statistical analysis**

- t-test, Chi-square test, Log-rank test, ANOVA test or Kruskal-wallis test

- HbA1c, C-peptide, lipid profile, Insulin, glucagon, IGF-1, GLP-1, Neuropeptide Y, Ghrelin, Leptin, HOMA scale: as an longitudinal data, comparing them by repeated measurement analysis of variance (RMANOVA) for comparing between Roux-en-Y gastrojejunostomy and Gastroduodenostomy

-two side p-value of less than 0.05 will be considered as statistical significance

1. Criteria for evaluation of safety, including side effects, evaluation methods and reporting methods

There are no changes in the existing surgical methods and data collection process, so there is no plan to report any postoperative complications or side effects.

1. Interim analysis

There is no planed interim analysis in this study.

1. Monitoring for data, process, and safety of this study

Investigator will do regular check.

1. Time table for the study

|  | **추진일정 (월)** | | | | | |
| --- | --- | --- | --- | --- | --- | --- |
|  | **0-1** | **2-13** | | **14-25** | **26-30** | **31-36** |
| **IRB Approval** |  |  | |  |  |  |
| **Patients’ enrollment** |  |  |  |  |  |  |
| **Data collection** |  |  |  |  |  |  |
| **Patients’ follow up** |  |  |  |  |  |  |
| **Analysis** |  |  |  |  |  |  |
| **Report** |  |  |  |  |  |  |

1. References

1) Joshua PT, David EC Cummings Hormonal and Metabolic Mechanisms of Diabetes Remission after Gastrointestinal Surgery. Endocrinology 2009 150:2518-2525

2) Korner J, Bessler M, Inabnet W, Taveras C, Holst JJ 2007 Exaggerated glucagon-like peptide-1 and blunted glucose-dependent insulinotropic peptide secretion are associated with Roux-en-Y gastric bypass but not adjustable gastric banding. Surg Obes Relat Dis 3:597–601

3) Cummings DE, Overduin J, Foster-Schubert KE, Carlson MJ 2007. Role of the bypassed proximal intestine in the anti-diabetic effects of bariatric surgery. Surg Obes Relat Dis 3:109–115

4) Rubino F, Gagner M, Gentileschi P, Kini S, Fukuyama S, Feng J, Diamond E. The early effect of the Roux-en-Y gastric bypass on hormones involved in body weight regulation and glucose metabolism. Ann Surg. 2004 Aug;240(2):236-42

5) Rubino F, Forgione A, Cummings DE, Vix M, Gnuli D, Mingrone G, Castagneto M, Marescaux J. The mechanism of diabetes control after gastrointestinal bypass surgery reveals a role of the proximal small intestine in the pathophysiology of type 2 diabetes. Ann Surg. 2006 Nov;244(5):741-9

6) Kojima K, Yamada H, Inokuchi M, Kawano T, Sugihara K. A comparison of Roux-en-Y and Billroth-I reconstruction after laparoscopy-assisted distal gastrectomy. Ann Surg. 2008 Jun;247(6):962-7.

7) Yang J, Li C, Liu H, Gu H, Chen P, Liu B. Effects of subtotal gastrectomy and Roux-en-Y gastrojejunostomy on the clinical outcome of type 2 diabetes mellitus. J Surg Res. 2010 Nov;164(1):e67-71. Epub 2010 Jul 30.
