# Supplementary material for: A randomized controlled trial of Roux-en-Y gastrojejunostomy vs. gastroduodenostomy with respect to the improvement of type 2 diabetes mellitus after distal gastrectomy in gastric cancer patients
Source: PLoS One. 2017 Dec 7;12(12):e0188904. doi: 10.1371/journal.pone.0188904 (PMC5720795; doi:10.1371/journal.pone.0188904)
Supplement: S3 File — The original protocol of this study which was approved by Institutional Review Board. (DOC) [file pone.0188904.s008.doc]

**임상연구 계획서**

**1) 연구 제목**

당뇨가 있는 위암 환자에서 위암 수술 후 재건 시 문합 방법에 따른 당뇨 치료 기전 및 효과 연구

**2) 연구 필요성 및 개요**

**제 2형 당뇨의 수술적 치료**

최근 제 2형 당뇨에 대한 치료로써 비만대사 수술이 새로운 치료 방법의 하나로 주목 받고 있다. 비만대사 수술은 처음에 비만 환자의 체중을 감소시키기 위한 목적으로 시행된 수술의 결과 비만 환자의 고혈당이 개선되는 것이 보고되면서 고혈당 등의 대사성 질환의 치료에 도입되었다.

대사 수술은 위장관 경로를 외과적으로 변경 또는 조절하는 수술이며 복강경 수술이 도입됨으로써 수술 흉터에 대한 부담이 적고 회복을 빠르게 할 수 있는 최소침습적 수술을 적용하고 있다. 환자의 상태에 따라 Adjustable gastric banding (AGB), Roux-en-Y gastric bypass (RYGB), biliopancreatic diversion (BPD) 등 수술 술식이 시행되고 있으며 혈당 조절의 효과가 뛰어난 Roux-en-Y gastric bypass, biliopancreatic diversion 가 주로 시행되고 있다.

Roux-en-Y 위우회술은 비만대사수술의 하나로도 흔히 시행되고 있으며 작은 위주머니를 만들어 Treitz 인대 하방 30~50 cm 하방의 소장을 절단하며 원위부 소장과 위를 연결하고 근위부 소장을 위공장문합부에서 50~150cm 하방에 연결하는 술식이다.


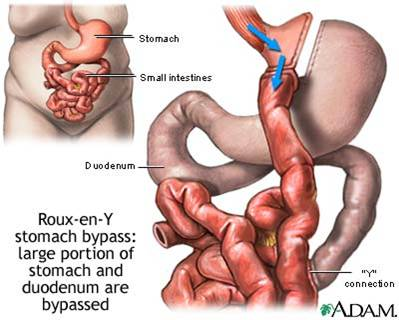


수술 후 80% 이상에서 고혈당이 개선되며 80% 가량에서 경구용 혈당 강하제 복용을 유의하게 감소시키고 79% 에서 인슐린 투여량을 감소시킨다고 보고되었다. 또한 체질량지수가 35 미만으로 비교적 낮은 환자들에서도 효과가 있었음이 확인 되었다.

**수술에 의한 고혈당 개선의 기전**

수술에 의한 고혈당 개선의 기전으로 음식 섭취량 및 흡수를 제한할 뿐 아니라 음식물이 근위부 소장을 우회하여 원위부 소장에 빨리 도달함으로써 혈당의 대사와 관련된 여러가지 호르몬의 분비에 변화를 유발하여 인슐인 내성 조절 및 혈당 개선 효과를 가져온다고 보고되었다.

여기에는 GLP-1, IGF-1, Leptin, CCK, Ghrelin, Neuropeptide Y 등 다양한 호르몬이 관여하고 있으며 우회 수술이 이러한 호르몬의 분비에 영향을 주어 고혈당을 개선시키게 된다.

**1) The upper intestinal hypothesis**

십이지장을 영양소가 통과하지 않게 되면서 상부 소장과 영양소가 접촉하지 않아 인슐린 내성에 관여된 비정상적인 호르몬 분비과정이 촉발되지 않아 고혈당 조절에 도움이 된다는 가설이다. Dr. Francesco Rubino는 이 가설을 뒷받침하기 위해 RYGB에서 stomach을 제외하지 않는 변형 수술법인 DJB(duodenal-jejunal bypass)를 고안해 내어, nonobese type 2 DM rat인 GK(Goto-Kakizaki) rat에 DJB를 시행한 결과 큰 체중 감소나 식사량 감소 없이도 DM이 매우 호전된 것을 관찰하였다. 십이지장을 소화 경로에 포함시키거나 제외한 여러 변형 수술을 시행했을 때 십이지장을 포함시키거나 제외함에 따라 DM이 재발되거나 호전되는 결과를 보인다는 보고도 있다. 이 이론을 뒷받침하는 추가적인 근거로 endoluminal duodenal sleeve를 이용해 duodenum의 mucosa에 음식물이 접촉하지 않도록 하였을 때 체중 감소량은 미미하지만 혈당량은 크게 감소하며 HbA1c level도 insulin 투여에 못지 않게 낮아지는 결과를 들 수 있다.


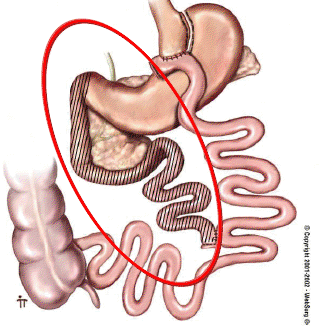


**Proximal bowel hypothesis** : 십이지장을 영양소가 통과하지 않게 되면서 인슐린 내성에 관여된 비정상적인 호르몬 분비과정이 촉발되지 않음. (Rubino et al. *Ann Surg, 2006)*

**2) The lower intestinal hypothesis**

RYGB와 BPD의 소장 우회 술식에 의해 영양분이 기존에 비해 원위부 소장에 더 많이 도착하게 되는데, 이에 따라 incretin peptide인 GLP-1의 분비가 촉진되는 것이 glucose homeostasis에 긍정적인 영향을 준다는 가설이다. GLP-1은 glucose-dependent insulin secretion을 촉진하고 glucagon secretion을 억제하며 gastric emptying을 지연시키는 효과를 가지고 있으며, 실험 동물에서 beta-cell mass를 증가시키기도 한다고 알려져 있다. GLP-1은 Peptide YY와 oxyntomodulin과 함께 ileum과 colon의 L-cell에서 영양분 자극에 의해 분비되어 식욕 억제와 혈당 감소 효과를 가져온다. 원위부 소장에 소화가 덜 진행된 영양소가 가장 많이 도착하는 경우는 BPD 수술로, bypass가 덜 극단적인 RYGB에 비해 BPD에서 GLP-1 증가가 더 확연히 관찰된다. 한편, GLP-1 분비는 영양분과의 직접 접촉뿐 아니라 십이지장에서의 신경 자극에 의해서도 촉진되므로 십이지장을 우회하는 RYGB에서는 GLP-1 분비가 저해될 가능성이 있으나, 최근 연구 결과에서는 RYGB에서도 충분한 GLP-1 증가가 일어나며 AGB에서는 그렇지 않음이 보고되었다


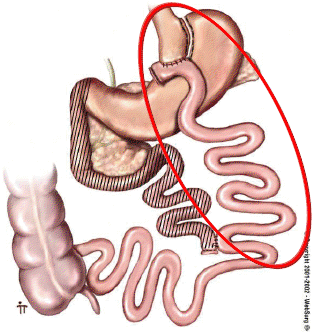


**Distal bowel hypothesis** : 원위부 소장에 음식물이 일찍 도착하면서, 해당 영역의 L-cell들이 GLP-1을 분비하도록 자극받아 glucagon 호르몬 작용이 촉진됨 (Rubino et al. *Ann Surg, 2006)*

**3) The ghrelin hypothesis**

Ghrelin은 식욕을 증가시키며 당뇨 악화와 관련된 상부위장관 호르몬으로, 식전에 급격히 증가하고 식후에 억제된다. 또한 평상시 혈중 순환 농도는 식이요법에 의해 감량된 체중과 비례하여, 체중 감량에 대항하는 호르몬이라 할 수 있다. Ghrelin은 90% 이상이 위와 십이지장에서 생성되며 RYGB는 이 곳을 우회하기 때문에, 이 수술법에 의해 ghrelin의 생성이 방해 받을 것이라 생각할 수 있다. 실제로 RYGB 수술을 받은 후 ghrelin 농도는 체중 감소에도 불구하고 낮아지는 것이 확인되었다. Ghrelin은 인슐린과 길항 작용을 하는 호르몬 분비를 촉진하고, insulin-sensitizing hormone인 adiponectin을 억제하며, 간에서 phosphatidylinositol-3-kinase signaling을 차단해 insulin signaling을 막는 등의 작용을 통해 혈당을 증가시키기 때문에, RYGB 등 수술을 통해 ghrelin을 감소시킬 수 있다면 그에 따라 혈당도 감소될 수 있을 것이다.

**당뇨를 동반한 위암환자의 수술적 치료**

위암의 경우 위절제 수술 후 환자들의 체중이 감소하면서 고혈압, 당뇨 등의 동반 질환이 개선되는 것을 경험하게 되는데 특히 당뇨가 동반되어 있는 위암 환자에서는 비만대사 수술의 술기를 접목시켜 고혈당을 개선시킬 수 있는 효과를 증가시킬 수 있을 것으로 생각된다.

현재 임상에서 시행되는 위암의 수술적 치료 방법은 원위부 위절제 및 림프절 곽청술을 시행하는 것이며 위절제 후 문합술은 당뇨 여부에 상관없이 종양의 위치나 수술자의 선호도에 따라 위십이지장 문합술, 위공장문합술, Roux-en-Y 위공장 문합술이 시행되고 있다. 문합법의 종류는 위암의 치료 결과에 영향을 미치지 않을 것으로 생각되며 세가지 문합법 모두 흔히 적용되는 술식이다. 또한 최근 발표된 소규모의 후향적 연구에서 당뇨를 동반한 위암 환자에서 위아전절제술 시행 후 Roux-en-Y 위공장 문합술을 시행한 경우 약 57%의 환자에서 당뇨치료제를 투여하지 않고도 혈당이 잘 조절되었다는 결과를 보고하였다.

그러므로 본 연구에서는 당뇨가 있는 위암 환자에서 십이지장을 우회하지 않는 위십이지장문합술과 십이지장 및 상부 공장을 우회하는 Roux-en-Y 위공장 문합술을 비교하여 Roux-en-Y 위공장 문합술이 당뇨 개선에 얼마나 효과가 있는지 확인하여 당뇨가 있는 위암 환자에서 술식을 선택하는데 도움이 되고자 한다.


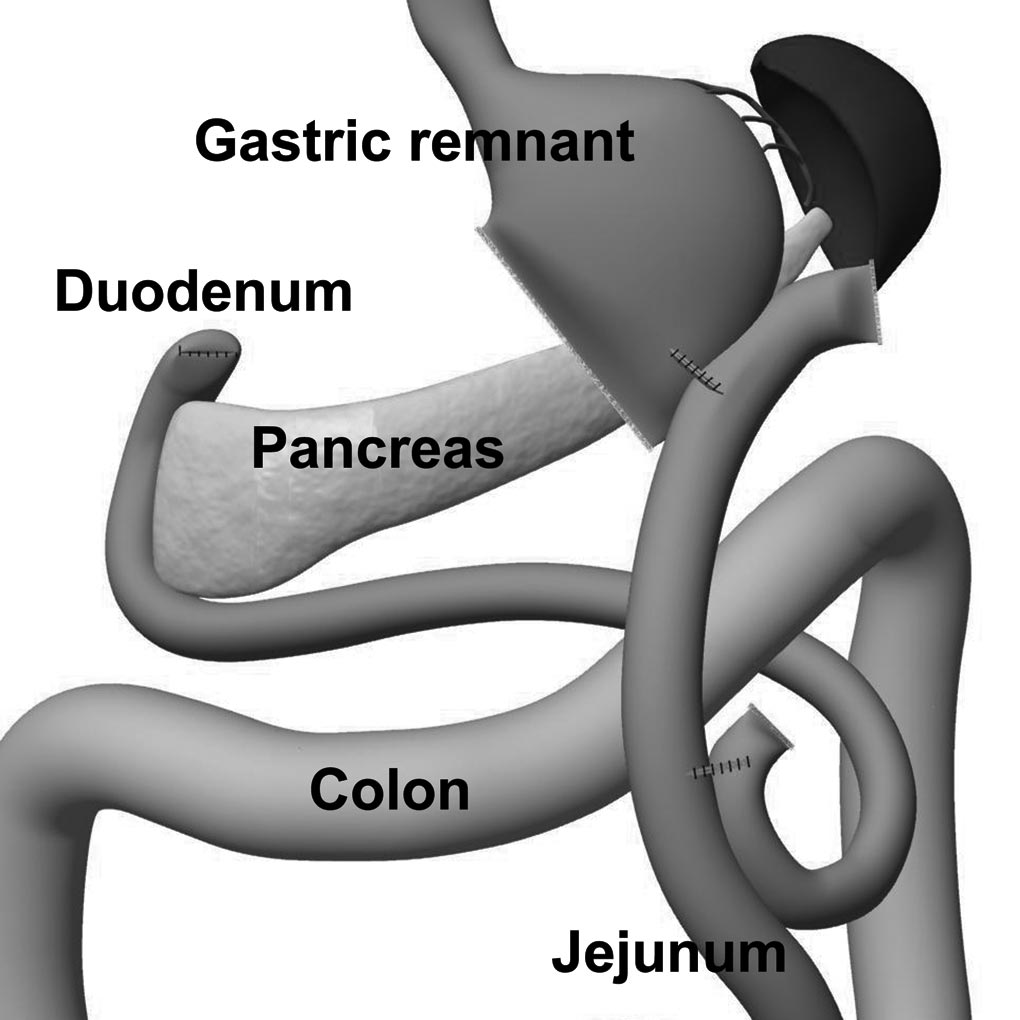

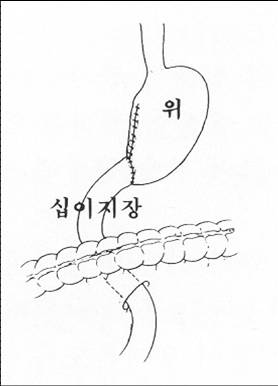


< Roux-en-Y 위공장 문합술) <위십이지장문합술>

** Roux-en-Y 위공장 문합술

표준 위절제술 및 림프절 곽청술 시행 후 Treitz 인대 하방 25~30cm 에서 공장을 절단한다.

근위부(원위부) 공장은 남은 위와 side-to-side 문합하고 위-공장문합부에서 30-40cm 하방에서 근위부 공장과 원위부 공장을 side-to-side 문합한다.

** 위-십이지장 문합술

표준 위절제술 및 림프절 곽청술 시행 후 원형 문합기를 이용하여 십이지장과 남은 위를 문합하고 선형 문합기를 이용하여 위를 봉합한다.

현재까지 위암 환자를 대상으로 시행된 무작위 전향적 연구는 없으며 이 연구의 결과는 위암의 치료에 있어, 현대의 주요 성인병 중 하나의 당뇨의 치료 효과까지 극대화 함으로써 환자의 삶의 질을 증가시키고 의료비 감소에 기여할 것으로 생각된다.

**3) 연구 목적**

1. 당뇨를 동반한 위암 환자에서 원위부 위절제술 시행 후 위십이지장문합술과 Roux-en-Y 위공장 문합술의 고혈당 개선 효과를 비교하여 당뇨환자에서 적절한 문합 술식을 결정하는데 도움이 되고자 한다.

2. 위암 수술 시 문합 방법에 따른 당뇨 관련 수치 및 호르몬의 변화를 관찰하고 고혈당 개선의 기전을 밝힌다.

**4).연구 수행 장소 및 기간**

장소 – 연세대학교 세브란스병원

기간 – 2011. 05.01 ~ 2014. 04.30 (36개월)

**5).피험자의 선정 또는 제외기준 및 스크리닝 검사 항목**

**선정기준**

1. 수술 전 검사 (상부위장관내시경,복부 CT scan) 상 조기위암으로 진단된 위암환자 중 당뇨로 진단되어 치료받고 있거나 수술 전 검사를 통해 당뇨로 확진 된 환자

2. 암의 중심부가 위의 하부에 위치해 있어 원위부 위아전절제술이 가능한 환자

3. 20~80세

4. 연구에 대해 이해하고 동의한 환자

**제외기준**

1. 타장기암 및 전신 염증성 질환으로 치료 받은 적이 있는 환자

2. 수술 전 조절되지 않는 중대한 동반질환이 있는 환자

3. 취약한 피험자 (의사능력결여, 임산부, 미성년자 등)

4. 수술이 필요한 합병증이 발생한 환자

**6).목표 피험자의 수 및 산출 근거**

본원의 후향적 연구 데이터에서 원위부 위아전절제술 시행 후 위-십이지장 문합술을 시행한 경우 수술 후 1년째에 당뇨약을 끊게 되는 환자의 percentage 가 15% 가량이며, 기존의 문헌 보고에서 의하면 Roux-en-Y 위공장 문합술 후 1년째에 당뇨약을 끊게 되는 환자의 percentage 가 57% 임을 고려하였을 때 각 군당 sample size는 18명, 총 36명이 필요함. 중도탈락율 10%를 고려하면 40명 정도임.

**Two Independent Proportions (Null Case) Power Analysis**

**Numeric Results of Tests Based on the Difference: P1 - P2**

**H0: P1-P2=0. H1: P1-P2=D1<>0. Test Statistic: Likelihood Ratio test**

**Sample Sample Prop|H1 Prop**

**Size Size Grp 1 or Grp 2 or Diff Diff**

**Grp 1 Grp 2 Trtmnt Control if H0 if H1 Target Actual**

**Power N1 N2 P1 P2 D0 D1 Alpha Alpha Beta**

0.8091 18 18 0.5700 0.1500 0.0000 0.4200 0.0500 0.0764 0.1909

Note: exact results based on the binomial were only calculated when both N1 and N2 were less than 100.

**References**

Chow, S.C.; Shao, J.; Wang, H. 2003. Sample Size Calculations in Clinical Research. Marcel Dekker. New York.

D'Agostino, R.B., Chase, W., Belanger, A. 1988.'The Appropriateness of Some Common Procedures for Testing the Equality of Two Independent Binomial Populations', The American Statistician, August 1988, Volume 42 Number 3, pages 198-202.

Fleiss, J. L., Levin, B., Paik, M.C. 2003. Statistical Methods for Rates and Proportions. Third Edition. John

Wiley & Sons. New York.

Lachin, John M. 2000. Biostatistical Methods. John Wiley & Sons. New York.

Machin, D., Campbell, M., Fayers, P., and Pinol, A. 1997. Sample Size Tables for Clinical Studies, 2nd

Edition. Blackwell Science. Malden, Mass.

**Report Definitions**

'Power' is the probability of rejecting a false null hypothesis. It should be close to one.

'N1 and N2' are the sizes of the samples drawn from the corresponding populations.

'P1' is the proportion for group one under H1. This is the treatment or experimental group.

'P2' is the proportion for group two. This is the standard, reference, or control group

'Target Alpha' is the probability of rejecting a true null hypothesis that was desired.

'Actual Alpha' is the value of alpha that is actually achieved.

'Beta' is the probability of accepting a false null hypothesis.

**Summary Statements**

Group sample sizes of 18 in group one and 18 in group two achieve 81% power to detect a difference between the group proportions of 0.4200. The proportion in group one (the treatment group) is assumed to be 0.1500 under the null hypothesis and 0.5700 under the alternative hypothesis. The proportion in group two (the control group) is 0.1500. The test statistic used is the two-sided Likelihood Ratio test. The significance level of the test was targeted at 0.0500. The significance level actually achieved by this design is 0.0764.

추정을 위해 사용된 식은 다음과 같다.


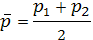


또한 본원의 후향적 조사에서 1,2기 위암으로 근치적 위아절제술을 시행 받은 환자 중 당뇨로 진단된 환자는 연간 50명 가량이므로 탈락자를 고려하여 1년간 약 40 명의 환자를 enroll 가능하므로 각각 20명씩 시험군과 대조군으로 나누어 연구를 진행하는데 무리가 없을 것으로 생각됨.

무작위 배정표는 다음과 같고 당뇨 유병 기간을 층화변수로 사용함.

| **OBS** | **당뇨** | |
| --- | --- | --- |
|  | **5년이상** | **5년 미만** |
| **1** | 1 | 2 |
| **2** | 2 | 1 |
| **3** | 2 | 2 |
| **4** | 1 | 1 |
| **5** | 2 | 2 |
| **6** | 1 | 1 |
| **7** | 1 | 2 |
| **8** | 2 | 1 |
| **9** | 1 | 2 |
| **10** | 2 | 1 |
| **11** | 1 | 2 |
| **12** | 2 | 1 |
| **13** | 2 | 2 |
| **14** | 1 | 1 |
| **15** | 1 | 2 |
| **16** | 2 | 1 |
| **17** | 1 | 1 |
| **18** | 2 | 2 |
| **19** | 2 | 1 |
| **20** | 1 | 2 |

이 연구를 통하여 문합 방법에 따라 당뇨 관련 수치들에 차이가 관찰 된다면 이를 바탕으로 앞으로 좀 더 대규모의 환자들을 대상으로 전향적 연구를 다시 시행할 계획임

**7)연구 설계 및 방법**

**<연구 설계>**

1. 0-1 개월 IRB 통과

2. 2-13개월 환자 등록 및 검사 시행

3. 14-25개월 추적 관찰

4. 26-36개월 결과 분석 및 보고

**<연구 방법>**

* 위암으로 위아전절제 수술 예정이면서 당뇨로 약 또는 인슐린을 투여 중 이거나, 수술 전 혈당 검사를 통해 당뇨병을 확진 한 환자를 대상으로 함.

* 수술 전 환자를 선택하여 무작위적으로 실험군과 대조군으로 나눈다. 눈가림 하지 않음.

실험군: Roux-en-Y 위공장문합술을 시행한 군

대조군: 위십이지장 문합술 시행군

* 주요 비교 항목: 수술 전, 수술 후 7일, 수술 후 3개월, 수술 후 1년째

체중, 공복 시 혈당, 식후 2시간 혈당, HbA1c, C-peptide(공복시/식후2시간), lipid profile, Insulin(공복시/식후2시간), glucagon, IGF-1, GLP-1, Neuropeptide Y, Ghrelin, Leptin, 등을 혈액에서 확인.

HOMA scale 분석 HI=plasma glucose (mmol/l)× insulin (UI/ml)/22.5

* 결과를 분석하여 당뇨가 있는 위암 환자에서 십이지장을 우회하지 않는 위십이지장 문합술에 비해 Roux-en-Y 위공장 문합술이 당뇨 개선에 효과가 있는지 확인

**8) 관찰항목·임상검사항목 및 관찰검사방법**

- 키, 몸무게

- 혈압, 맥박 측정

- 당뇨 유병기간, 당뇨 치료기간, 치료제, 치료제 용량

- 투여중인 인슐린의 종류와 용량에 대한 조사

- 음주, 흡연 유무 여부

- 수술 전 진단 결과, 병리 소견, 수술 후 경과 및 합병증, 사망 발생 유무 및 세부사항

- 체중, 혈액검사 (일반혈액검사, 화학검사, 빈혈검사, 염증반응검사 등의 기본검사), 공복 시 혈당, 식후 2시간 혈당, HbA1c, C-peptide, lipid profile, Insulin, glucagon, IGF-1, GLP-1, Neuropeptide Y, Ghrelin, Leptin, HOMA scale

: 수술 전, 수술 후 1주일, 수술 후 3개월, 수술 후 1년째 측정

- 같은 시기별로 삶의 질 관련 설문지 조사

(일반 위암 환자와 동일한 일정의 외래 방문)

***호르몬 수치 분석**

1) 혈액 채취 및 혈장 분리

수술 전날과 수술 후 7일, 3개월, 1년째 혈액 수집예정

8시간 가량의 금식 상태에서 혈액을 채취하여 EDTA(ethylenediamine tetra-acetic acid) 용기에 수집한 후 4℃, 1600 x g 에서 15분간 원심분리기로 혈장을 분리하여 실험 전까지 -70℃에 보관한다.

2) radioimmunoassay (RIA) 시행

분리된 혈장에서 다음과 같은 commercial RIA kits를 사용하여 각 호르몬의 양을 분석한다.

<commercial RIA kits>

Ghrelin과 Leptin (Linco Research, St. Charles, Missouri)

GLP-1과 NPY (Interscience Institute, Inglewood, CA)

Glucagon, IGF-1 (Endocrine Sciences Inc., Calabasas, CA)

**9) 예측 부작용 및 사용상의 주의사항**

- 특이사항 없음

- 일반적인 위암 절제술 후 부작용과 동일

**10) 임상연구 중지·탈락 기준**

- 피험자의 임상 참여 동의 철회 시

**11) 유효성 평가기준, 평가방법 및 해석방법(통계분석방법)**

**평가 기준**

- 수술 후 당뇨 관련 혈액 수치의 개선

- 당뇨 치료제 투여 횟수 및 용량 감소

**해석방법**

- t-test, Chi-square test, Log-rank test, ANOVA test or Kruskal-wallis test

- HbA1c, C-peptide, lipid profile, Insulin, glucagon, IGF-1, GLP-1, Neuropeptide Y, Ghrelin, Leptin, HOMA scale 의 level 등을 수술 전, 수술 후 1주일, 수술 후 3개월, 수술 후 1년째 측정하여

이를 Roux-en Y group과 BI group 사이에 차이가 있는지, 각 group내에서 수술 전, 후, 3개월, 1년 경과 후 level에 차이가 있는지에 대하여 repeated measurement ANOVA(MANOVA) 로 확인

- P<0.05이하일 경우 효과가 있다고 판단

**12) 부작용을 포함한 안전성의 평가기준, 평가방법 및 보고방법**

- 기존의 수술 방법 및 자료 수집과정에서 변경된 것이 없으므로 수술 후 합병증 또는 부작용 발생 시 보고 할 계획 없음

**13) 중간분석에 대한 계획 및 필요 시 연구의 조기 종료하는 범위를 포함하여 연구의 통계 분석에 대한 계획 기술**

- 중간 분석 계획 없음

**14) 연구의 목적을 위해 적용되는 의약품 또는 기타 시술의 지속적인 안전성을 모니터링하는 계획과 필요 시 이런 목적의 독립적인 자료 모니터링(자료 및 안전성 모니터링) 위원회의 지정**

- 연구자가 수시로 모니터링 할 계획입니다.

**15) 연구실행 계획표** (일정표)

|  | **추진일정 (월)** | | | | | |
| --- | --- | --- | --- | --- | --- | --- |
|  | **0-1** | **2-13** | | **14-25** | **26-30** | **31-36** |
| **IRB승인** |  |  | |  |  |  |
| **환자등록** |  |  |  |  |  |  |
| **전향적 데이터 수집** |  |  |  |  |  |  |
| **등록환자 추적** |  |  |  |  |  |  |
| **데이터분석** |  |  |  |  |  |  |
| **결과보고** |  |  |  |  |  |  |

**16) 참고문헌**

1) Joshua PT, David EC Cummings Hormonal and Metabolic Mechanisms of Diabetes Remission after Gastrointestinal Surgery. *Endocrinology* 2009 150:2518-2525

2) Korner J, Bessler M, Inabnet W, Taveras C, Holst JJ 2007 Exaggerated glucagon-like peptide-1 and blunted glucose-dependent insulinotropic peptide secretion are associated with Roux-en-Y gastric bypass but not adjustable gastric banding. *Surg Obes Relat Dis* 3:597–601

3) Cummings DE, Overduin J, Foster-Schubert KE, Carlson MJ 2007. Role of the bypassed proximal intestine in the anti-diabetic effects of bariatric surgery. *Surg Obes Relat Dis* 3:109–115

4) Rubino F, Gagner M, Gentileschi P, Kini S, Fukuyama S, Feng J, Diamond E. The early effect of the Roux-en-Y gastric bypass on hormones involved in body weight regulation and glucose metabolism. Ann Surg. 2004 Aug;240(2):236-42

5) Rubino F, Forgione A, Cummings DE, Vix M, Gnuli D, Mingrone G, Castagneto M, Marescaux J. The mechanism of diabetes control after gastrointestinal bypass surgery reveals a role of the proximal small intestine in the pathophysiology of type 2 diabetes. Ann Surg. 2006 Nov;244(5):741-9

6) Kojima K, Yamada H, Inokuchi M, Kawano T, Sugihara K. A comparison of Roux-en-Y and Billroth-I reconstruction after laparoscopy-assisted distal gastrectomy. Ann Surg. 2008 Jun;247(6):962-7.

7) Yang J, Li C, Liu H, Gu H, Chen P, Liu B. Effects of subtotal gastrectomy and Roux-en-Y gastrojejunostomy on the clinical outcome of type 2 diabetes mellitus. J Surg Res. 2010 Nov;164(1):e67-71. Epub 2010 Jul 30.
